# Enterovirus A71 does not meet the uncoating receptor SCARB2 at the cell surface

**DOI:** 10.1371/journal.ppat.1012022

**Published:** 2024-02-15

**Authors:** Yorihiro Nishimura, Kei Sato, Yoshio Koyanagi, Takaji Wakita, Masamichi Muramatsu, Hiroyuki Shimizu, Jeffrey M. Bergelson, Minetaro Arita

**Affiliations:** 1 Department of Virology II, National Institute of Infectious Diseases, Musashimurayama-shi, Tokyo, Japan; 2 Division of Infectious Diseases, The Children’s Hospital of Philadelphia, Philadelphia, Pennsylvania, United States of America; 3 Laboratory of Viral Pathogenesis, Institute for Virus Research, Kyoto University, Sakyo-ku, Kyoto, Japan; 4 Department of Infectious Disease Research, Institute of Biomedical Research and Innovation, Foundation for Biomedical Research and Innovation at Kobe, Kobe-shi, Hyogo, Japan; 5 Department of Pediatrics, University of Pennsylvania Perelman School of Medicine, Philadelphia, Pennsylvania, United States of America; National Institute of Allergy and Infectious Diseases, UNITED STATES

## Abstract

Enterovirus A71 (EV-A71) infection involves a variety of receptors. Among them, two transmembrane protein receptors have been investigated in detail and shown to be critical for infection: P-selectin glycoprotein ligand-1 (PSGL-1) in lymphocytes (Jurkat cells), and scavenger receptor class B member 2 (SCARB2) in rhabdomyosarcoma (RD) cells. PSGL-1 and SCARB2 have been reported to be expressed on the surface of Jurkat and RD cells, respectively. In the work reported here, we investigated the roles of PSGL-1 and SCARB2 in the process of EV-A71 entry. We first examined the expression of SCARB2 in Jurkat cells, and detected it within the cytoplasm, but not on the cell surface. Further, using PSGL-1 and SCARB2 knockout cells, we found that although both PSGL-1 and SCARB2 are essential for virus infection of Jurkat cells, virus attachment to these cells requires only PSGL-1. These results led us to evaluate the cell surface expression and the roles of SCARB2 in other EV-A71–susceptible cell lines. Surprisingly, in contrast to the results of previous studies, we found that SCARB2 is absent from the surface of RD cells and other susceptible cell lines we examined, and that although SCARB2 is essential for infection of these cells, it is dispensable for virus attachment. These results indicate that a receptor other than SCARB2 is responsible for virus attachment to the cell and probably for internalization of virions, not only in Jurkat cells but also in RD cells and other EV-A71–susceptible cells. SCARB2 is highly concentrated in lysosomes and late endosomes, where it is likely to trigger acid-dependent uncoating of virions, the critical final step of the entry process. Our results suggest that the essential interactions between EV-A71 and SCARB2 occur, not at the cell surface, but within the cell.

## Author summary

Enterovirus A71 (EV-A71) is a major pathogen of hand-foot-and-mouth disease. Although a variety of receptor molecules have been proposed to mediate EV-A71 infection, one of them, SCARB2, is widely believed to serve as the primary attachment and entry receptor on many cell types. SCARB2—initially identified as a lysosomal integral membrane protein, LIMP II—is primarily expressed within the cytoplasm rather than on the cell surface. In this study, we examined how this largely cytoplasmic protein functions during EV-A71 attachment and infection. In contrast to earlier reports, we found that SCARB2 was not expressed at detectable levels on the surface of EV-A71–susceptible cell lines, and that its elimination with CRISPR/Cas9 had no effect on attachment of virions to the cell surface. Nonetheless, SCARB2 was readily detected on late endosomes and lysosomes, and is essential for productive infection. The results suggest that non-SCARB2 receptors are responsible for virus attachment and internalization, whereas SCARB2 within late endosomes and lysosomes is an intracellular receptor essential for a subsequent event in infection.

## Introduction

Enterovirus A71 (EV-A71) is a non-enveloped single-stranded RNA virus that belongs to the species *Enterovirus A* of the genus *Enterovirus* in the family *Picornaviridae* (for a recent review of EV-A71, see [1]). EV-A71 is a major pathogen of hand-foot-and-mouth disease, most commonly a mild illness in young children. In some cases, however, EV-A71 infection sometimes has severe manifestations, including flaccid paralysis, brainstem encephalitis, pulmonary edema, and even death [2,3]. EV-A71 outbreaks have been frequent in the Asia-Pacific region since the end of the 20^th^ century [4,5], and outbreaks of severe EV-A71 neurological disease occurred in Spain in 2016 [6] and in the United States in 2018 [7].

**Fig 1:**
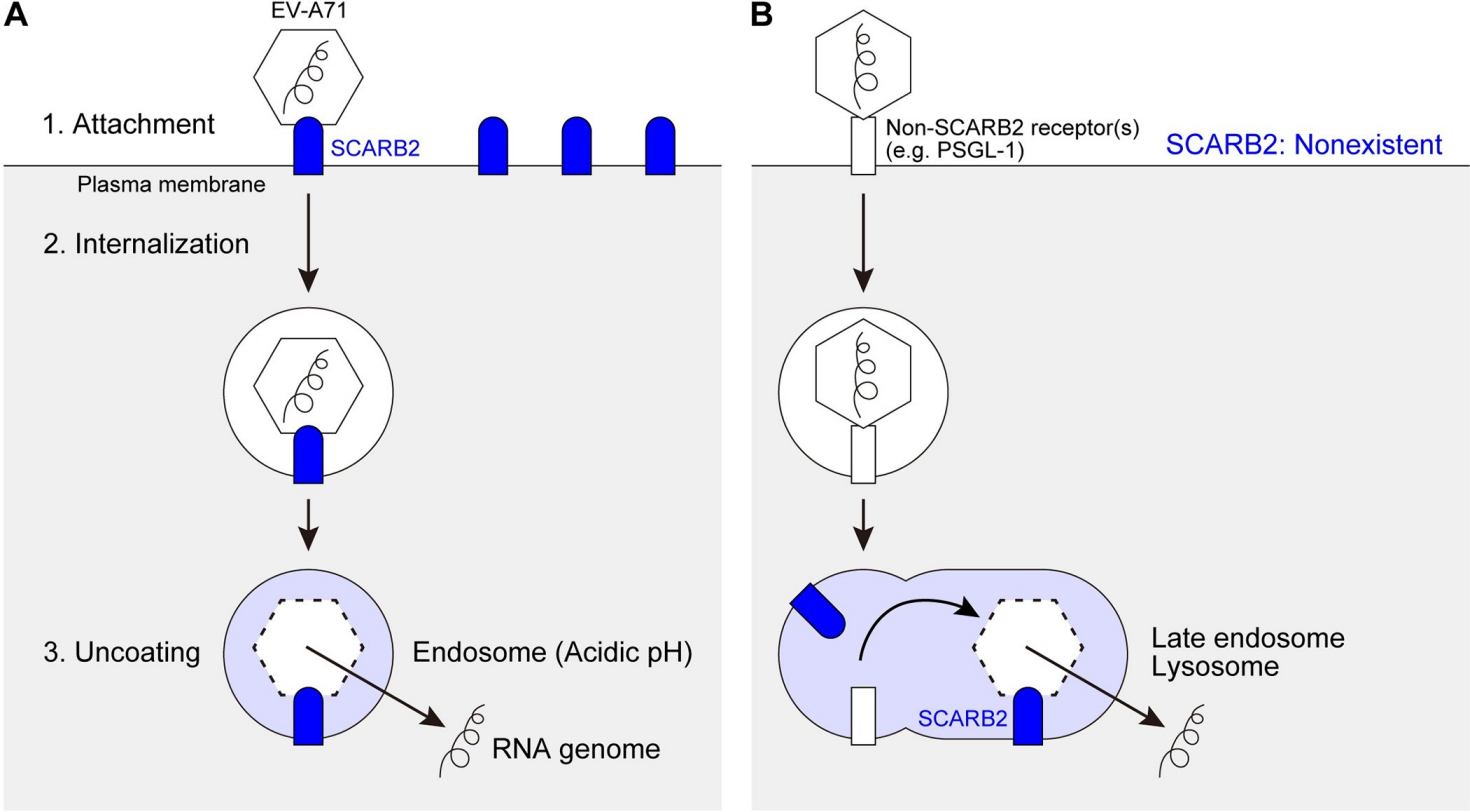
Two models of the EV-A71 entry. (A) The previous model suggested by Yamayoshi et al. [9,23]. SCARB2 is highly expressed on the cell surface. Surface SCARB2 binds to EV-A71 at the south rim of the canon [24] and initiates viral entry in endosomal vesicles. SCARB2 induces uncoating when the vesicle lumen becomes acidic. (B) Our new model. SCARB2 is absent from the cell surface. A non-SCARB2 receptor mediates EV-A71 attachment, most likely interacting with the viral five-fold vertex [20,21]. Virus is internalized and transported through the endosomal system, meeting SCARB2 within late endosomes or lysosomes, where uncoating occurs.

A virus initiates cell entry by binding to a receptor on the cell surface. We previously reported that some isolates of EV-A71 bind to the P-selectin glycoprotein ligand-1 (PSGL-1) to infect Jurkat T cells and other haematopoietic cell lines [8]. Contemporaneously with our work, another EV-A71 receptor—the scavenger receptor class B member 2 (SCARB2)—was identified from rhabdomyosarcoma (RD) cells commonly used for EV-A71 isolation [9] (Fig 1A). A variety of additional molecules have been proposed as potential attachment or entry receptors for EV-A71 [10–18].

Among the EV-A71 receptors identified so far, PSGL-1 and SCARB2 are best characterized at the molecular level [19–24]. SCARB2—also called lysosomal integral membrane protein (LIMP) II [25]—functions in the delivery of β-glucocerebrosidase from the endoplasmic reticulum to the lysosome [26], as well as in cholesterol traffic through lysosomes [27]. Expression of human SCARB2 in murine cells [9] or in transgenic mice [28,29] permits infection by EV-A71, clearly demonstrating its importance to infection. In vitro, EV-A71 interaction with soluble SCARB2 (particularly under acidic conditions) initiates the uncoating process by which viral RNA is released into the cell [23,30]. Because SCARB2 is highly concentrated in lysosomes and late endosomes [31], it is ideally located to mediate uncoating, which is known to depend on endosomal acidification [32]. Recent work suggests that EV-A71 uncoating occurs within mature late endosomes [33].

PSGL-1 is primarily expressed on haemopoietic cells, where it mediates cell adhesion to vascular endothelium [34,35]. EV-A71 interaction with PSGL-1 is essential for attachment and infection in Jurkat cells and other hematopoietic cell lines [8]. But unlike SCARB2, PSGL-1 does not promote uncoating in vitro [23,30]. We hypothesized that in Jurkat cells, infection requires attachment to PSGL-1 at the cell surface, but that uncoating depends on a subsequent intracellular interaction with SCARB2 (Fig 1B). In the experiments reported here, we confirmed that PSGL-1 is the major EV-A71–binding receptor on the surface of Jurkat cells, as we reported previously. Further, we found that although SCARB2 is necessary for infection of Jurkat cells, it is neither expressed on the cell surface nor involved in EV-A71 attachment. These results led us to examine the localization and function of SCARB2 in infection of other EV-A71–susceptible cells.

## Results

### Jurkat cells don’t express SCARB2 on the cell surface

First, we examined SCARB2 expression on Jurkat cells. We previously showed that EV-A71 infection is inhibited by anti-PSGL-1 antibody (Ab), indicating that PSGL-1 is the primary EV-A71 receptor on Jurkat cells [8]. In addition, we also demonstrated that the amino acid at VP1-145 at the five-fold vertex of the capsid determines EV-A71 binding to PSGL-1 and infectivity in Jurkat cells [20]. Namely, EV-A71 isolates with VP1-145G/Q bind to PSGL-1 and efficiently infects Jurkat cells, but those with VP1-145E do not [20]. Nonetheless, VP1-145 does not affect virus interaction with SCARB2, which binds to the south rim of the canyon far away from the five-fold vertex [24]. Therefore, we would expect that if SCARB2 is expressed on the surface of Jurkat cells, and interaction with SCARB2 leads to infection, EV-A71 with VP1-145E should efficiently infect Jurkat cells, even though it does not bind to PSGL-1.

We thus speculated that SCARB2 is not expressed on the surface of Jurkat cells. To address this hypothesis, we employed two anti-SCARB2 Abs: first, a goat polyclonal Ab (pAb) (R&D systems, Cat# AF1966) used in the previous study to detect SCARB2 on the surface of RD cells [9]; and second, a rabbit monoclonal Ab (mAb) (clone 12H5L1, Invitrogen, Cat # 702770). The specificity of the anti-SCARB2 Abs was validated by immunoblotting against recombinant soluble SCARB2 fused to the immunoglobulin Fc region (SCARB2-Fc), using SCARB1-Fc as a negative control (S1 Fig).

**Fig 2:**
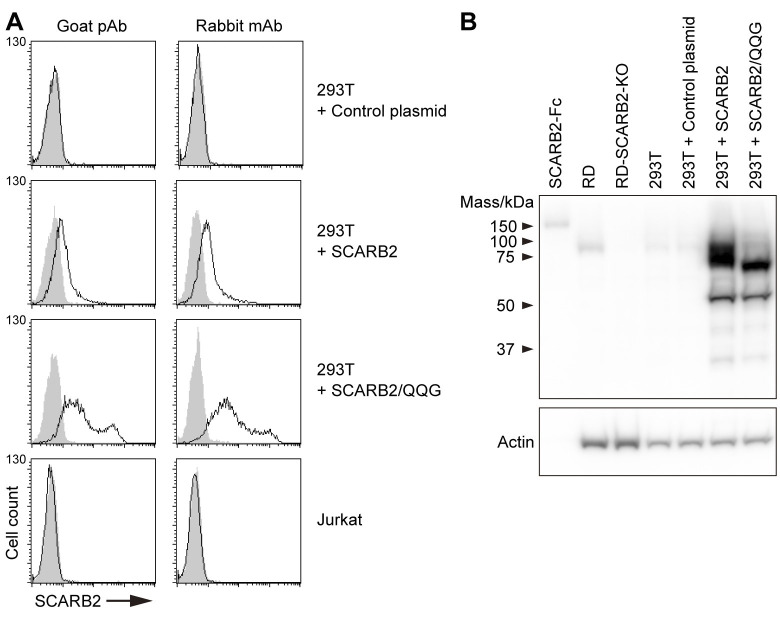
Jurkat cells do not express SCARB2 on the cell surface. (A) Flow cytometric analysis of cell-surface SCARB2 using goat pAb and rabbit mAb (clone 12H5L1). To confirm both Abs are applicable to flow cytometry, 293T cells overexpressing SCARB2 or mutant SCARB2 with the three amino acid substitutions to enhance cell-surface expression (SCARB2/QQG) and Jurkat cells were stained in parallel. The solid line and the shaded area represent staining with anti-SCARB2 Ab and control IgG, respectively, followed by Alexa Fluor 488-tagged secondary Ab. The figure is representative of three independent experiments. (B) A large amount of overexpressed SCARB2 existed inside the 293T cells. Western blotting analysis with anti-SCARB2 mAb (clone 12H5L1). Recombinant SCARB2-Fc (5 ng) was loaded as a positive control. RD and RD-SCARB2-KO (clone No.3) cells were loaded as positive and negative controls, respectively. The figure is representative of three independent experiments.

To confirm that the SCARB2 Abs can detect cell-surface SCARB2 by flow cytometry, we examined 293T cells transiently transfected with SCARB2 expression plasmids or with a control plasmid (Fig 2). No SCARB2 was detected with either Ab on the surface of 293T control transfectants (Fig 2A), although these cells did express SCARB2 detectable by immunoblot of whole-cell lysates (Fig 2B). In cells transfected with a SCARB2 plasmid, a marked increase in whole-cell SCARB2 expression was evident by immunoblot, whereas only small amounts of SCARB2 on the cell surface were detected, as demonstrated by rightward shifts in the flow cytometry histogram.

The vast majority of SCARB2, a resident lysosomal protein, is known to be retained in the lysosomal compartment rather than delivered to the plasma membrane [31,36]. Surface expression of SCARB2 is enhanced by disruption of a lysosome-targeting signal in the C-terminal cytoplasmic region (I476G) [31]; mutation of specific glycosylation sites (N68Q and N325Q) within the ectodomain also prevents SCARB2 targeting to lysosomes [37,38]. We introduced N68Q, N325Q, and I476G into SCARB2 to enhance expression at the cell surface. When the triple mutant (SCARB2/QQG) was overexpressed in 293T cells, marked rightward shifts in the fluorescence histograms were seen with both the polyclonal goat and monoclonal rabbit Abs. This result indicated that both anti-SCARB2 Abs can be used to detect SCARB2 on the cell surface by flow cytometry. Nonetheless, when these Abs were used to stain Jurkat cells, no SCARB2 was detected on the cell surface (Fig 2A; Jurkat).

### Anti-SCARB2 Ab does not inhibit EV-A71 infection of Jurkat cells

**Fig 3:**
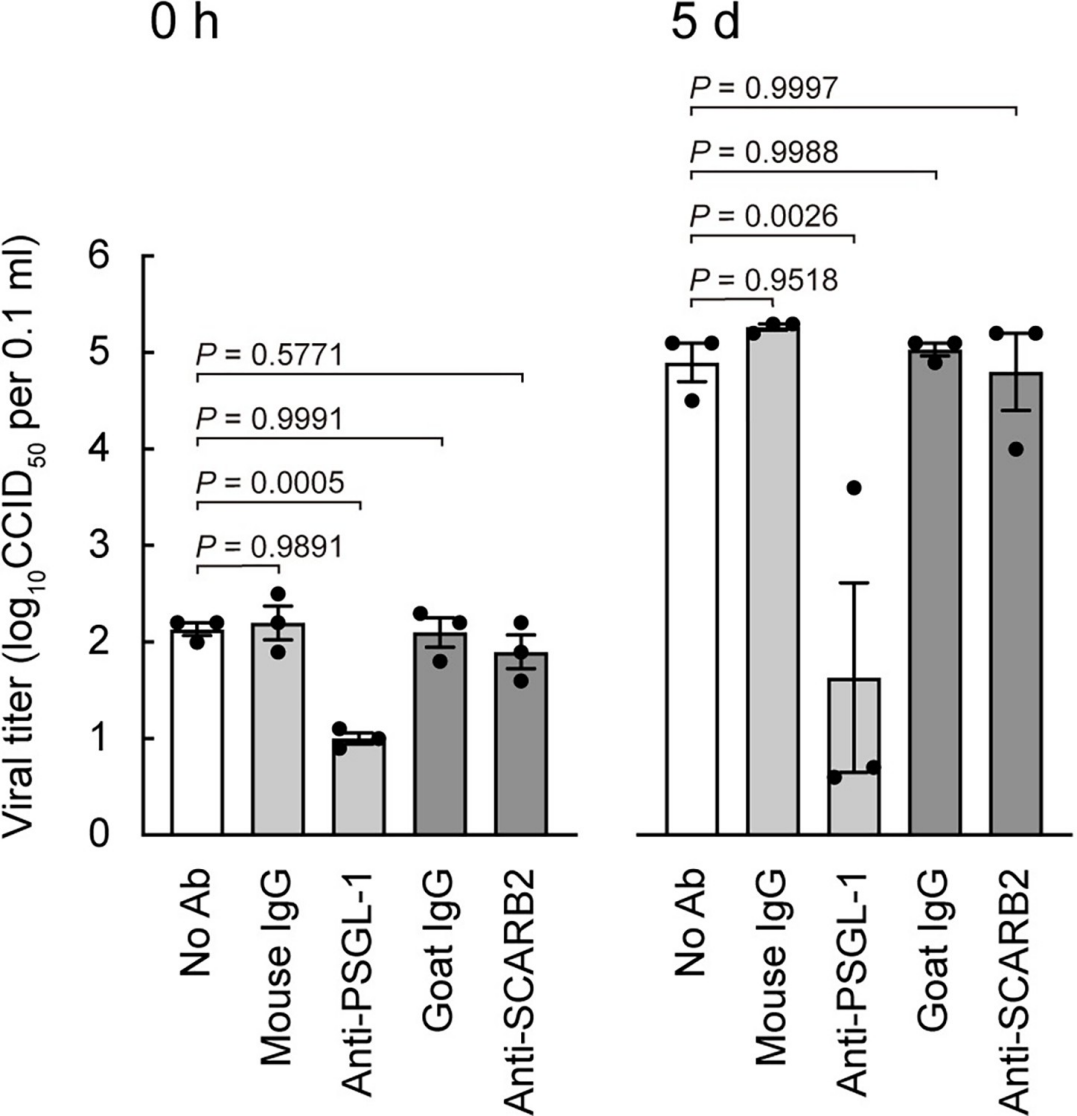
Anti-SCARB2 pAb does not inhibit EV-A71 binding to and replication in Jurkat cells. Jurkat cells were pre-treated with anti-PSGL-1 mAb (10 μg ml^-1^), anti-SCARB2 pAb (50 μg ml^-1^), or control Abs on ice for 1 h, followed by infection with EV-A71 at 1 CCID_50_ per cell on ice for 1 h. Cells were then washed (0 h), cultured without Abs, and harvested at 5 days (5 d) post-infection. Results are indicated as the mean and s.e. for triplicate samples.

To evaluate the functions of PSGL-1 and SCARB2 in EV-A71 interaction with Jurkat cells, we examined the effect of anti-PSGL-1 mAb or anti-SCARB2 pAb on viral infection (Fig 3). We employed the PSGL-1–binding strain of EV-A71, SK-EV006 [39], which was used in the previous study [9]. Jurkat cells were pre-treated with each Ab, then exposed to EV-A71 on ice for 1 h; cells were washed and immediately frozen (0 h) for measurement of viral binding, or cultured for 5 days (in the absence of additional Ab) to permit replication, then viral titers were determined. Titers at 0 h, which reflected virus binding to the cell surface, were significantly reduced by anti-PSGL-1, but not by anti-SCARB2 or control Abs. Titers at 5 days were also reduced by anti-PSGL-1 but not by anti-SCARB2 or control Abs. Thus anti-PSGL-1 but not anti-SCARB2 Ab inhibited both virus binding and infection in Jurkat cells. Although anti-SCARB2 pAb had no effect on virus interaction with Jurkat cells, it nonetheless was capable of inhibiting virus attachment to isolated SCARB2, prepared as an Fc fusion protein (S2 Fig). These results indicate that SCARB2 is not responsible for EV-A71 attachment to Jurkat cells.

### EV-A71 needs both surface PSGL-1 and internal SCARB2 to infect Jurkat cells

**Fig 4:**
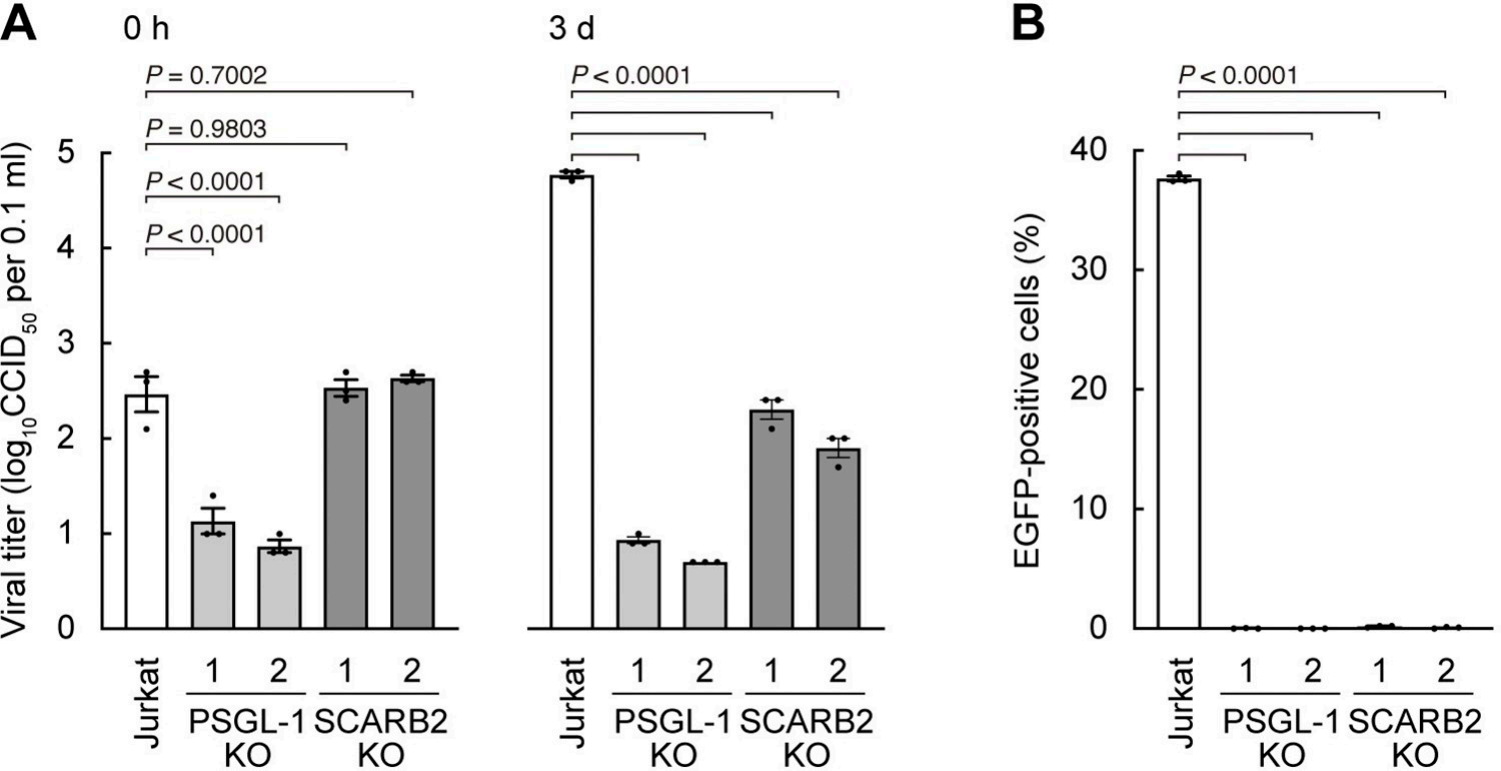
SCARB2 is not involved in EV-A71 binding to Jurkat cells, but necessary for viral replication. PSGL-1 or SCARB2 were knocked out by CRISPR/Cas9 in Jurkat cells, and two each of clones were established. (A) Cells were infected with EV-A71 at 1 CCID_50_ per cell on ice for 1 h, then washed (0 h), cultured, and harvested at 3 days (3 d) post-infection. (B) Cells were infected with EV-A71-EGFP at 10 CCID_50_ per cell and cultured for 18 h. Infected cells were identified by flow cytometry to detect EGFP expression. Results are indicated as the mean and s.e. for triplicate samples.

To examine the role of PSGL-1 and SCARB2 in EV-A71 infection more strictly, we used the CRISPR/Cas9 system [40] to knock out the expression of either PSGL-1 or SCARB2 in Jurkat cells (S3 Fig). To establish genetically uniform clones, we first selected a Jurkat clone which is highly susceptible to EV-A71 infection. Using the cloned Jurkat cells, we established two clones each of knockout (KO) cells: PSGL-1-KO and SCARB2-KO clones. Effective KO of PSGL-1 and SCARB2 was confirmed by flow cytometry for PSGL-1 (S3A Fig) and western blotting for SCARB2 (S3B Fig). To evaluate EV-A71 binding and infectivity in Jurkat cells and the KO clones, cells were infected with EV-A71 on ice for 1 h. Then the cells were washed and frozen (0 h) or washed and cultured for three days. The viral titer at 0 h, a measure of virus attachment, was significantly decreased in the PSGL-1-KO clones but not in the SCARB2-KO clones (Fig 4A). After cultivation for three days, the viral titer increased substantially in wild-type Jurkat cells, but not in either PSGL-1-KO or SCARB2-KO clones. Thus, knocking out of PSGL-1 inhibited both virus attachment and infection, whereas knocking out of SCARB2 inhibited virus infection with no effect on virus attachment. Taken together, the results indicate that, at least in Jurkat cells, SCARB2 functions primarily at a post-attachment step in infection.

To further test the roles of PSGL-1 and SCARB2 in EV-A71 infection of Jurkat cells, we examined the replication of EV-A71-EGFP, a PSGL-1–binding isolate (SK-EV006 [39]) engineered to express enhanced green fluorescent protein (EGFP) in infected cells, which had been used in the previous study [9] (Fig 4B). EV-A71-EGFP was added to Jurkat, PSGL-1-KO, and SCARB2-KO cultures and incubated for 18 h. Then infection was measured by flow cytometry to detect EGFP expression. Consistent with the results shown above (Fig 4A), EGFP expression was almost completely inhibited in either PSGL-1-KO or SCARB2-KO clones (Fig 4B). To eliminate the possibility of off-target effects of CRISPR/Cas9, we confirmed that re-expression of PSGL-1 (S4 Fig) or of SCARB2 fused to mCherry (S5 Fig) restored EV-A71-EGFP infectivity. These results confirmed that EV-A71 needs both PSGL-1 on the surface and SCARB2 within the cytoplasm to establish viral replication in Jurkat cells.

### SCARB2 is not expressed on the surface of several EV-A71–susceptible cell lines

**Fig 5:**
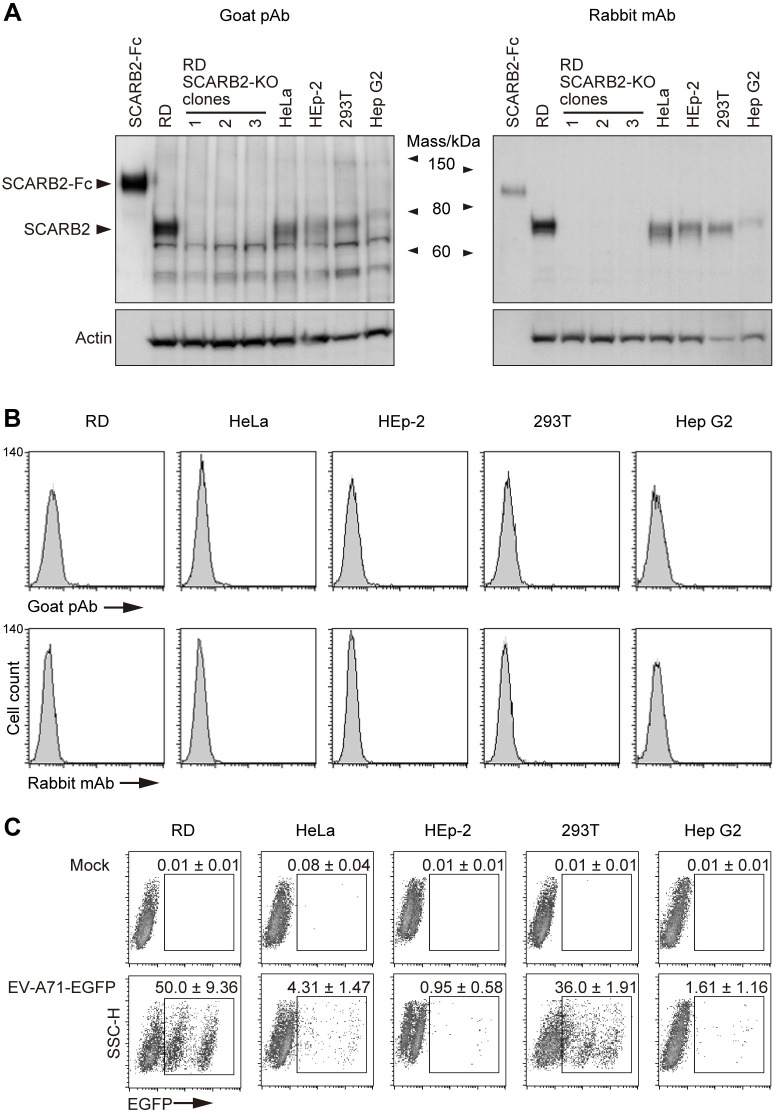
SCARB2 is absent from the cell surface, irrespective of EV-A71 susceptibility. RD, HeLa, HEp-2, 293T, and Hep G2 were obtained from the ATCC specifically for this study and used after limited passage. (A) Western blotting analysis by anti-SCARB2 pAb (left) and mAb (right, clone 12H5L1). Recombinant SCARB2-Fc (1 ng for pAb, 5 ng for mAb) was loaded as a positive control. RD-SCARB2-KO clones were loaded as negative controls. The figure is representative of three independent experiments. (B) Flow cytometric analysis by anti-SCARB2 pAb (top panels) and mAb (bottom panels), followed by Alexa Fluor 488-tagged secondary Ab. The solid line and the shaded area represent staining with anti-SCARB2 Ab and control Ab, respectively. Note that the solid line and the border of the shaded area are almost completely overlapped, indicating the absence of SCARB2 on the cell surface. Representative results with the following passage numbers after receiving from the ATCC; for pAb: RD, 3; HeLa, 3; HEp-2, 3; 293T, 3; Hep G2, 5; for mAb: RD, 13; HeLa, 5; HEp-2, 4; 293T, 4; Hep G2, 8. As a positive control of SCARB2 staining, cells expressing surface SCARB2 were always stained and analyzed in parallel. The figure is representative of three independent experiments. (C) EGFP expression in cells infected with EV-A71-EGFP. Cells were infected with EV-A71-EGFP at 10 CCID_50_ per cell and cultured for 18 h. Then EGFP expression was measured by flow cytometry. The EGFP-negative cells are not infected. The majority of EGFP-dim cells were infected early in the incubation period, and are dying and losing EGFP expression; some may have just been infected and are starting to express EGFP. The EGFP-bright cells were infected late in the incubation period are actively producing EGFP. The number indicates the percentage of EGFP-positive cells (mean and s.e. for three independent experiments).

Yamayoshi et al. [9] reported high levels of surface-SCARB2 expression on RD and HeLa cells, and lower levels on HEp-2c, 293T, and Hep G2 cells as determined by flow cytometric analysis; they also stated that the levels of SCARB2 at the cell surface correlated with susceptibility of these cell lines to EV-A71 infection [9]. As shown above, we found that EV-A71 replicated well in Jurkat cells that do not express detectable SCARB2 on the surface (Figs 2–4). We therefore hypothesized that there might be no correlation between the amount of surface SCARB2 and EV-A71 susceptibility. To directly address this hypothesis, we obtained the cell lines used in the previous study [9] from the ATCC and measured their expression of SCARB2 after only limited passage. First, we examined SCARB2 expression in whole cell lysates (Fig 5A) by immunoblotting with goat anti-SCARB2 pAb and with rabbit anti-SCARB2 mAb; as a negative control, we used three clones of RD-SCARB2-KO cells established using CRISPR/Cas9. With both Abs, SCARB2 was detected in RD, HeLa, HEp-2, 293T, and Hep G2 cells, but not in the RD-SCARB2-KO clones.

The previous study used goat anti-SCARB2 pAb to detect SCARB2 by flow cytometry [9], and we found that this pAb, as well as a rabbit anti-SCARB2 mAb, recognizes SCARB2 overexpressed on the surface of transfected cells (Fig 2). In contrast to the reported results [9], we did not detect SCARB2 on the surface of any of these cell lines either with the polyclonal goat or the monoclonal rabbit Abs (Fig 5B). We also found that SCARB2 was undetectable on the surface of RD-A cells, an RD-derived cell line passaged about two hundred thirty times and obtained from Centers for Disease Control and Prevention, USA (S6 Fig) (RD-A cells provided by our laboratory were used in [9]. Satoshi Koike: the corresponding author of [9], personal communication, 2023). Although the five cell lines did not express detectable SCARB2 at the cell surface, RD and 293T cells allowed efficient replication of EV-A71-EGFP, and the other cell lines permitted replication at a lower level (Fig 5C). Thus, we found that EV-A71 infected and replicated in multiple cell lines despite the absence of SCARB2 on the cell surface.

### SCARB2 is localized to late endosomes and lysosomes in RD cells

**Fig 6:**
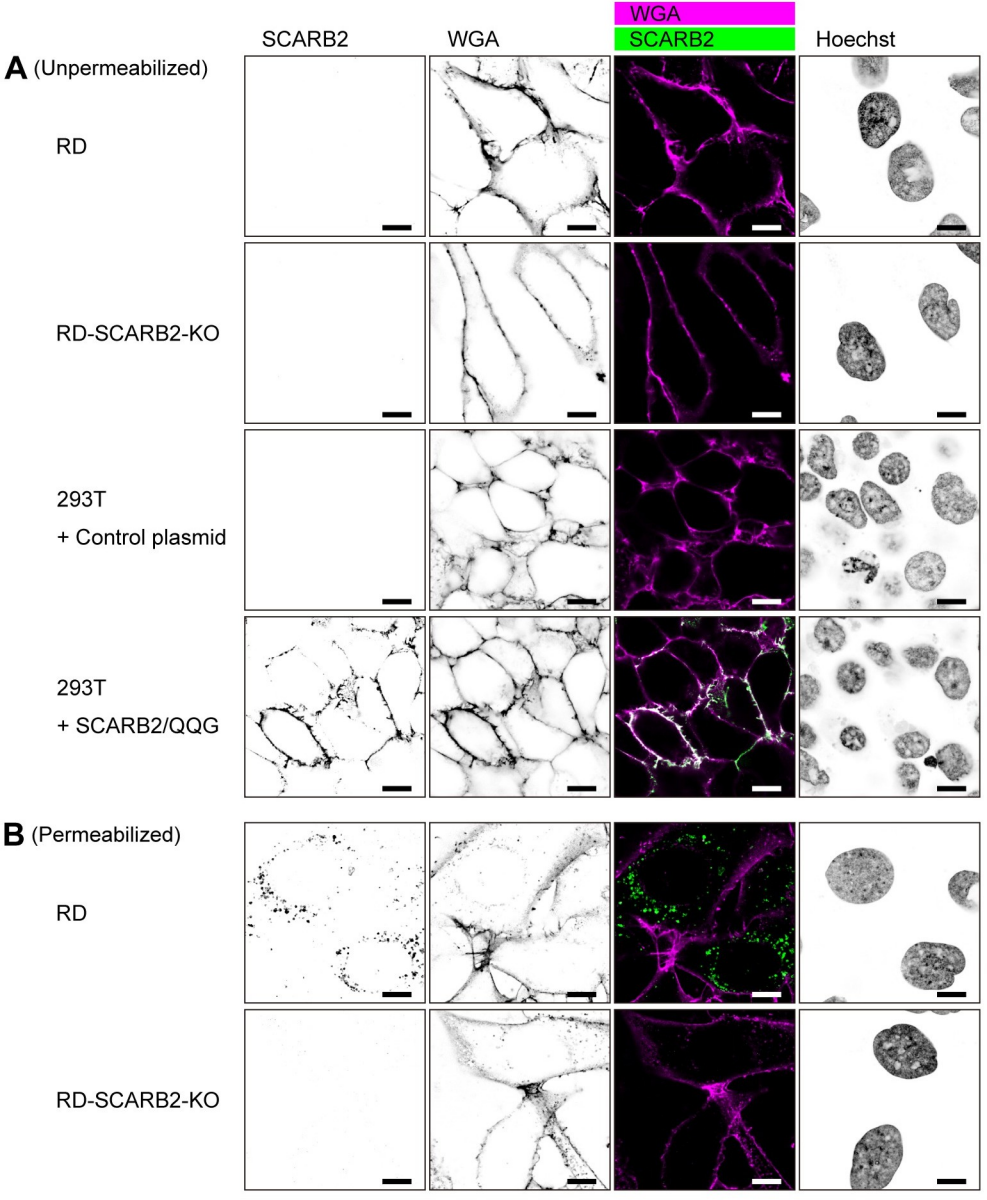
SCARB2 is absent from the cell surface and localized in the cytoplasm of RD cells. (A) Cells were stained with anti-SCARB2 mAb (clone 22H6L14) followed by Alexa Fluor 488-tagged secondary Ab and WGA conjugated with Alexa Fluor 633 without permeabilization. WGA was used to visualize the plasma membrane. Then the cells were fixed and observed under a confocal microscope. RD-SCARB2-KO cells (clone No.3) and 293T cells transfected with a control plasmid were used as negative controls. 293T cells expressing SCARB2/QQG on the cell surface were used as positive control. The figure is representative of three independent experiments. (B) RD and RD-SCARB2-KO (clone No.3) cells were stained with WGA, fixed, permeabilized, and stained with anti-SCARB2 mAb (clone 22H6L14) followed by Alexa Fluor 488-tagged secondary Ab. The figure is representative of three independent experiments. Scale bars, 10 μm.

**Fig 7:**
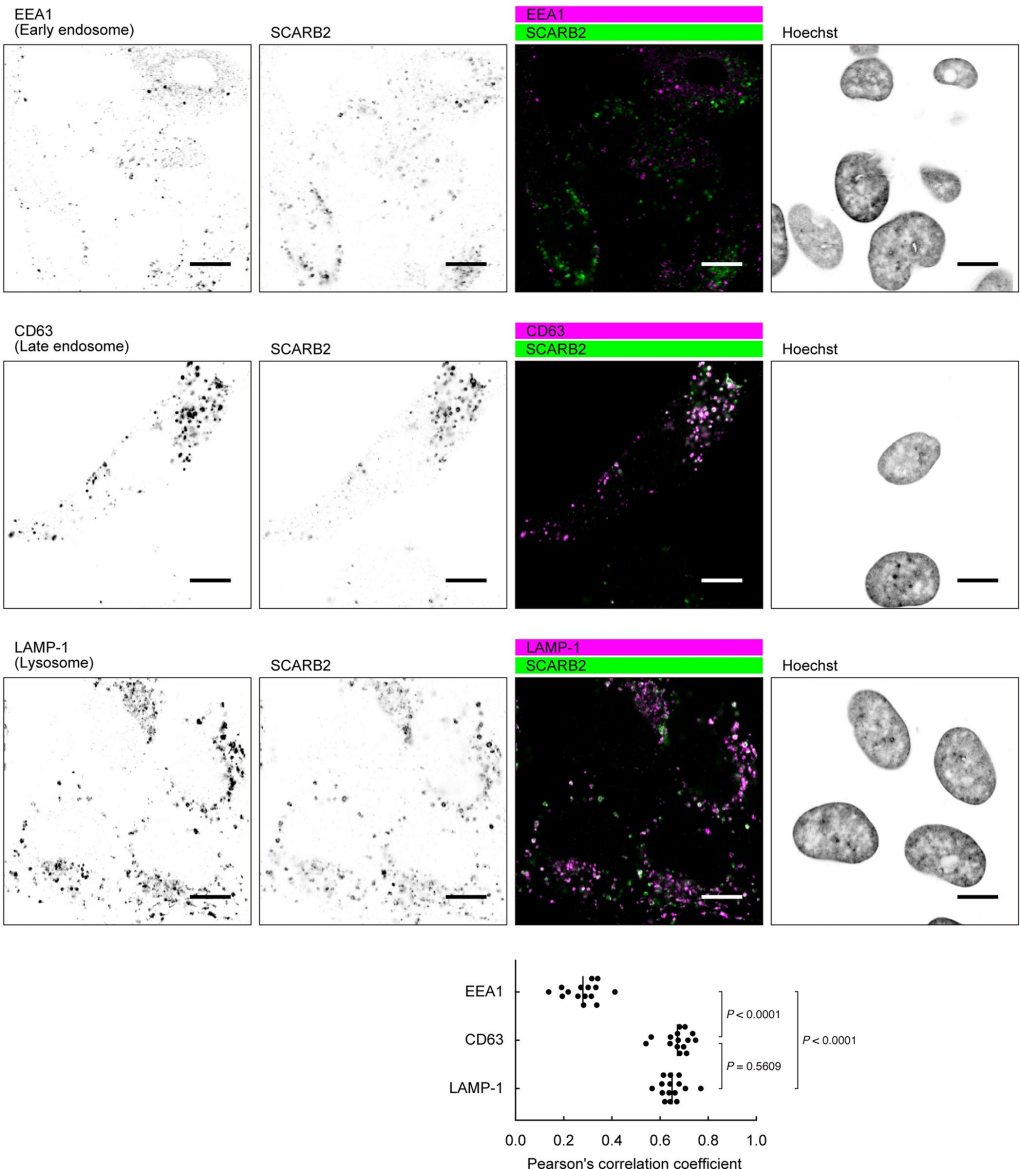
SCARB2 co-localizes with the markers of late endosomes and lysosomes in RD cells. RD cells were fixed, permeabilized, and stained with anti-SCARB2 mAb (clone 22H6L14) and mAb against either EEA1 (early endosome), CD63 (late endosome), or LAMP-1 (lysosome), followed by Alexa Fluor-tagged secondary Ab. Then the cells were observed under a confocal microscope. The figure is representative of three independent experiments. In each experiment, five pairs of images were analyzed. The graph shows colocalization between the markers and SCARB2 expressed as Pearson’s correlation coefficient. The vertical line indicates the mean value. Scale bars, 10 μm.

To examine the subcellular localization of SCARB2 in RD cells, we used confocal microscopy (Figs 6 and 7). For these experiments, we used a rabbit mAb (clone 22H6L14, Invitrogen, Cat # 703037) because we found that goat anti-SCARB2 pAb showed non-specific intracellular staining in RD-SCARB2-KO cells fixed with 4% paraformaldehyde (PFA) (S7 Fig), and because the rabbit mAb (clone 12H5L1) used in Figs 2, 5A, 5B and S3B did not detect SCARB2 in cells fixed with 4% PFA; the specificity of clone 22H6L14 was confirmed by immunoblotting with SCARB2-Fc (S1 Fig). In unpermeabilized RD cells, we did not detect SCARB2 at the plasma membrane, although plasma membrane staining was evident in 293T cells expressing SCARB2/QQG, used as a positive control (Fig 6A). After permeabilization, SCARB2 was easily detected in vesicles within the cytoplasm of RD cells (Fig 6B), but this staining of vesicles was not detected in RD-SCARB2-KO cells. SCARB2 is known to localize to late endosomes and lysosomes [26,36,41] where SCARB2-mediated uncoating is likely to occur. Consistent with this, SCARB2 in RD cells showed co-localization with the late endosomal marker CD63 and the lysosomal marker LAMP-1, but limited co-localization with the early endosomal protein EEA1 (Fig 7).

### Anti-SCARB2 Ab does not inhibit EV-A71 infection of RD cells

**Fig 8:**
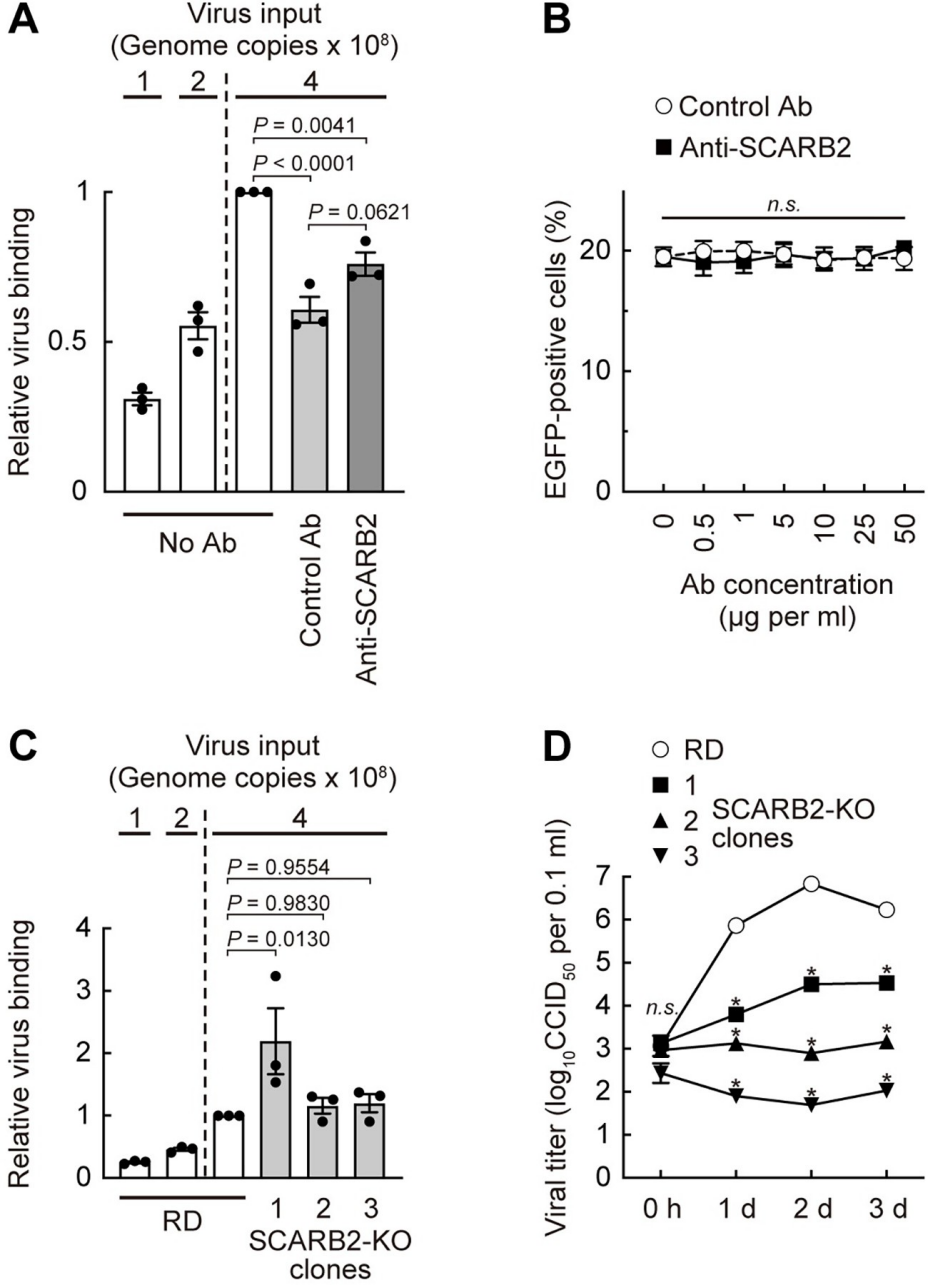
SCARB2 is not involved in EV-A71 binding to RD cells, but necessary for viral replication. (A) EV-A71 binding to RD cells in the presence of anti-SCARB2 pAb. RD cells pretreated with the anti-SCARB2 pAb (50 μg ml^-1^) were reacted with EV-A71 (4 × 10^8^ genome copies) on ice for 30 min. Then the cells were washed, and cellular and viral nucleotides were extracted. EV-A71 bound to the cell were analyzed by real-time RT-PCR by ΔΔCt method using *ATP5F1* mRNA as an endogenous control. As a technical control of detection of reduced copy number, quarter (1 × 10^8^ genome copies) and half (2 × 10^8^ genome copies) amount of EV-A71 was tested in parallel. The relative virus binding of RD cells reacted with 4 × 10^8^ genome copies of EV-A71 without Ab was expressed as 1. (B) EGFP expression in cells infected with EV-A71-EGFP in the presence of anti-SCARB2 pAb at 18 h post-infection. (C) EV-A71 binding to RD and RD-SCARB2-KO clones. EV-A71 bound to the cell were analyzed as in (A). The relative virus binding of RD cells reacted with 4 × 10^8^ genome copies of EV-A71 was expressed as 1. (D) Replication kinetics of EV-A71 in RD and RD-SCARB2-KO clones. Statistical significance was measured for each time point. Results are indicated as the mean and s.e. for three independent experiments (A, B, C) or triplicate analyses (D). Asterisks indicate *P* < 0.0001.

The previous study showed that EV-A71 attachment to RD cells was blocked by pretreatment of cells with anti-SCARB2 pAb in a dose-dependent manner [9] and suggested that SCARB2 functions as the major receptor for virus attachment. We previously found that a small compound, NF449, which blocks EV-A71 attachment to isolated PSGL-1 and to heparan sulfate (another attachment receptor of EV-A71), but does not inhibit attachment to isolated SCARB2, nonetheless blocked attachment to RD cells [21], suggesting that attachment to these cells might not depend on SCARB2. Our failure to detect SCARB2 on the surface of RD cells was consistent with the idea that another cell surface molecule is important for virus attachment. Nonetheless, in an effort to repeat the observations of Yamayoshi et al. [9], we determined whether the goat anti-SCARB2 pAb used by those authors—and found by us to inhibit virus attachment to isolated SCARB2 (S2 Fig)—blocked EV-A71 binding to RD cells (Fig 8A). Compared to the control Ab, anti-SCARB2 pAb had no inhibitory effect on virus attachment. This result was consistent with the idea that virus attachment to RD cells involves a receptor other than SCARB2.

We also examined whether the anti-SCARB2 pAb inhibited EV-A71-EGFP infection in a dose-dependent manner, as reported in the previous study [9] (Fig 8B). RD cells were pretreated with anti-SCARB2 pAb at several concentrations at 37°C for 30 min, then exposed to EV-A71-EGFP diluted in medium containing the same concentration of the anti-SCARB2 pAb, and incubated at 37°C for 18 hours. Even at the highest Ab concentration examined in the previous study [9], the percentage of EGFP-positive cells was not affected at all by the anti-SCARB2 pAb. Taken together, these results indicated the anti-SCARB2 pAb did not inhibit EV-A71 binding to, or infection of, RD cells, consistent with our observation that RD cells do not express SCARB2 on the cell surface (Figs 5B and 6A).

### EV-A71 binds efficiently to RD-SCARB2-KO cells, but replicates poorly

As a strict test of the role of SCARB2 in virus attachment, we measured virus binding to RD and RD-SCARB2-KO cells, and found that virus bound equally well to wild-type and KO cell lines (Fig 8C). (In fact, one of the KO clones bound somewhat more virus than did wild-type.) To determine whether other viral isolates also bound to cells in a SCARB2-independent manner, we examined four additional strains of EV-A71 with different PSGL-1 binding phenotypes (S8 Fig). These strains bound as well (or slightly better) to RD-SCARB2-KO cells as they did to wild-type RD cells. These results, consistent with an earlier report [42] that SCARB2 shRNA reduced infection without affecting attachment, confirm that SCARB2 has little, if any, role in EV-A71 binding to RD cells. In contrast, EV-A71 replication was markedly reduced in the RD-SCARB2-KO clones (Figs 8D and S9); in a control experiment, susceptibility to infection was restored to KO cells after transfection with a SCARB2 expression plasmid (S10 Fig), eliminating the possibility of off-target effects of CRISPR/Cas9. Taken together, the results with RD-SCARB2-KO cells confirm that SCARB2 is not required for virus attachment to RD cells, but is instead essential for a post-attachment step in infection.

### EV-A71 binds human primary cells in a SCARB2-independent manner

**Fig 9:**
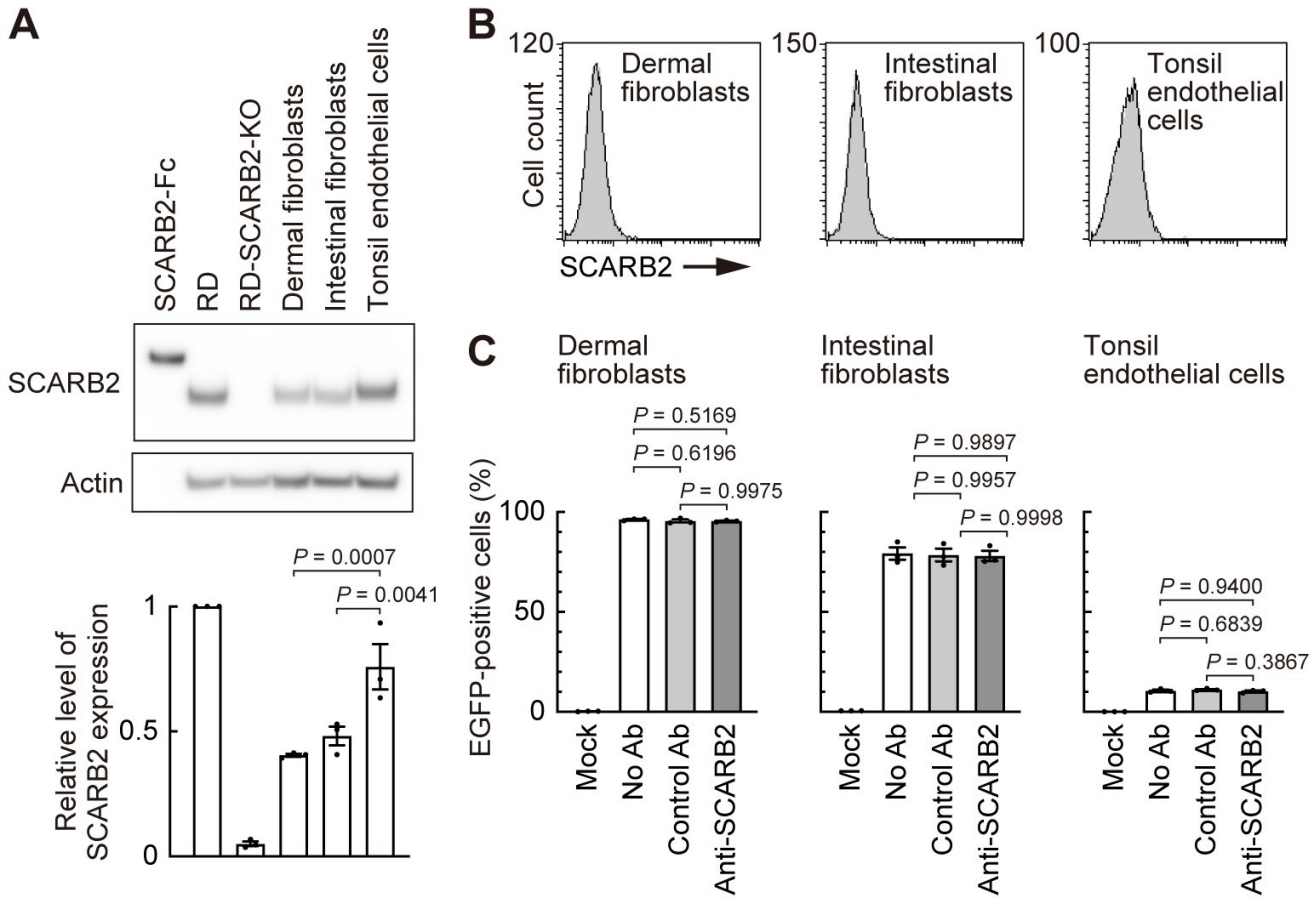
EV-A71 enters human primary cells in a SCARB2-independent manner. Human dermal fibroblasts (neonatal), intestinal fibroblasts, and tonsil endothelial cells were examined as cells presumed to be involved in the in vivo pathogenesis of EV-A71 infection. (A) Western blotting analysis with anti-SCARB2 mAb (clone 12H5L1). Recombinant SCARB2-Fc (5 ng) was loaded as a positive control. RD and RD-SCARB2-KO (clone No.3) cells were loaded as positive and negative controls, respectively. The figure is representative of three independent experiments. The graph displays the relative level of SCARB2 expression normalized by actin. The relative amount of SCARB2 in RD cells was expressed as 1. (B) Flow cytometric analysis by anti-SCARB2 pAb, followed by Alexa Fluor 488-tagged secondary Ab. The solid line and the shaded area represent staining with anti-SCARB2 pAb and control Ab, respectively. Note that the solid line and the border of the shaded area are almost completely overlapped, indicating the absence of SCARB2 on the cell surface. Representative results of cells passaged twice after receiving from the company. As a positive control of SCARB2 staining, cells expressing surface SCARB2 were always stained and analyzed in parallel. The figure is representative of three independent experiments. (C) EGFP expression in cells infected with EV-A71-EGFP in the presence of anti-SCARB2 pAb at 18 h post-infection. Cells with the following passage numbers after receiving from the company were used; dermal fibroblasts, 4; intestinal fibroblasts, 4; tonsil endothelial cells, 2. Results are indicated as the mean and s.e. for three independent experiments (A, C).

Finally, we examined the role of SCARB2 in EV-A71 infection of human primary cells (Fig 9). SCARB2 was detected in human dermal fibroblasts (neonatal), intestinal fibroblasts, and tonsil endothelial cells by immunoblot (Fig 9A). Consistent with our results using cell lines, SCARB2 was undetectable on the surface of these primary cells by flow cytometry (Fig 9B). As expected, EV-A71 infection of these primary cells was not inhibited by anti-SCARB2 pAb (Fig 9C). Interestingly, tonsil endothelial cells exhibited low EV-A71 infectivity despite abundant expression of SCARB2 detectable by immunoblot (Fig 9A). Thus, SCARB2 is not expressed on the surface of both human cell lines and primary cells we examined and EV-A71 most likely enters these cells in a SCARB2-independent manner.

## Discussion

Since the identification of SCARB2 as an EV-A71 receptor in 2009, it has been generally believed that SCARB2 is highly expressed on the surface of cells susceptible to infection, and that it mediates three important events in virus entry—attachment to the cell surface, internalization of virions, and uncoating within an endosomal compartment (Fig 1A). The results we report here are not consistent with this generally accepted view. We were unable to detect SCARB2 on the surface of susceptible cells and we found that anti-SCARB2 Ab had no inhibitory effect on virus binding or infection. Further, we found that SCARB2-KO using CRISPR/Cas9 had no effect on virus attachment to cells. These results indicate that SCARB2 is not important for virus attachment to the cell, and are not consistent with a role for SCARB2 as the primary attachment receptor for EV-A71.

Nonetheless, we found that SCARB2-KO prevents virus replication both in RD cells and in Jurkat cells, consistent with a variety of evidence supporting an essential role in infection for SCARB2. Given that SCARB2 is highly expressed within late endosomes and lysosomes, and is known to promote virus uncoating in an acidic environment, we propose a revised model of EV-A71 entry in which virus interacts with two distinct receptors (Fig 1B). First, EV-A71 binds to a primary non-SCARB2 receptor (or receptors), such as PSGL-1, on the cell surface. Then, virus enters the cell and moves through the endosomal compartment, encountering SCARB2 within acidic late endosomes or lysosomes where uncoating occurs.

Although some lysosomal membrane proteins are sorted indirectly, moving first to the plasma membrane and then reaching lysosomes only after endocytosis [43], several studies indicate that SCARB2 is directly targeted from the endoplasmic reticulum to lysosomes, and suggest that it reaches the cell surface primarily when its normal targeting to lysosomes is perturbed [31,36,44–46]. We found that even when highly overexpressed in 293T cells, the amount of wild-type SCARB2 delivered to the surface was quite small (Fig 2). Only when specific mutations (SCARB2/QQG) interrupted normal targeting did we detect SCARB2 at the cell surface at high levels. Thus, it seems extremely difficult to bring SCARB2 on the cell surface under physiological conditions.

Our data do not conflict with the observation that SCARB2 binds directly to virus [9,24]. We cannot exclude the possibility that SCARB2 is present at the cell surface at levels so low as to be undetectable by flow cytometry, but none of the cells we tested showed the high levels of surface SCARB2 reported in the previous study [9]. It is conceivable that because of rapid recycling, SCARB2 appears briefly at the cell surface, then is internalized so rapidly that it does not accumulate to detectable levels. Nonetheless, the results obtained with SCARB2-KO cells indicate that SCARB2 is not required for attachment of virions to the cell.

While we were preparing this paper, we became aware of recent work by Guo et al. [47], who performed a genome-wide CRISPR/Cas9 screen to identify genes required for EV-A71 infection in HeLa cells. These included SCARB2 itself; B3GAT3 and XYLT2, enzymes important for proteoglycan synthesis; and SLC35B2, a transporter of 3′-phosphoadenosine 5′-phosphosulfate, a sulfuryl donor essential for sulfation of both proteoglycans and protein tyrosine residues. Focusing on the general role of SLC35B2 in sulfation of host cells, Guo et al. [47] found that both sulfation of heparan sulfate and tyrosine sulfation of SCARB2 are involved in EV-A71 infection. They showed that knocking out either SLC35B2 or B3GAT3 prevented virus attachment, but that knocking out SCARB2 did not prevent binding or internalization of virions into RD and HeLa cells. These authors did not examine the expression of SCARB2 on the cell surface. Although we have some technical concerns about their experiments (in particular, their reported use of a membrane-avid dye, 1,1′-dioctadecyl-3,3,3′,3′-tetramethylindocarbocyanine perchlorate, to label a non-enveloped virus for entry studies) our data are consistent with their conclusion that SCARB2 is not involved in virus attachment to cells or in the subsequent entry of virions into the cell.

We previously found that EV-A71 interaction with Jurkat cells depends on tyrosine sulfation of PSGL-1 [19]. We also found that a highly sulfated small molecule, NF449, which has no effect on interaction with SCARB2, blocks virus attachment to PSGL-1 and heparan sulfate and prevents attachment to RD cells [21]. These results are consistent with the role of sulfation identified by Guo et al., and with the potential function of heparan sulfate as a primary attachment receptor on RD cells, as suggested by several groups [14,47,48]. In RD cells, attachment to heparan sulfate or another sulfated receptor is followed by internalization of virions occurs through clathrin-mediated endocytosis [32]; in Jurkat cells, attachment to sulfated PSGL-1 leads to internalization in caveolar vesicles [49]. When endosomes mature into late endosomes or fuse with lysosomes containing SCARB2, EV-A71 encounters SCARB2 under acidic conditions, and their interaction prompts the uncoating process (Fig 1B).

The virus entry process entails a series of events that begin with attachment of a virion to the cell surface and culminates in the release of genomic RNA into the cytoplasm (reviewed in [50,51]). In many cases—as is seen for poliovirus interaction with the receptor PVR—a single molecule mediates the entire process. In other cases, viruses may bind to cell surface molecules that do not themselves promote uncoating: for example, CD55 binds coxsackievirus B3 at the cell surface, but uncoating and infection require interaction with CAR [52]; similarly, many echoviruses bind CD55, but it was recently shown that interaction with the neonatal Fc receptor is essential for uncoating and infection to proceed [53,54]. It has become common to refer to attachment receptors and uncoating receptors. Since the identification of PSGL-1 and SCARB2 in 2009, a number of EV-A71–interacting molecules been proposed as putative attachment receptors but only SCARB2 is known to trigger the conformational changes associated with uncoating [55]. Given its intracellular localization, SCARB2 differs from cell surface enterovirus receptors like PVR and CAR. Instead, it resembles molecules such as NPC1 and LAMP1—so called intracellular receptors for hemorrhagic fever viruses—which promote viral fusion with endolysosomal membranes, but do not mediate virus attachment to cells [56].

SCARB2 has been considered by many investigators to be the primary EV-A71 receptor, in part based on evidence that it was highly expressed on the surface of RD cells and other susceptible permissive cell lines, and that infection was blocked by anti-SCARB2 Ab [9]. Although we have made every effort to ensure that we used reagents identical to those used by Yamayoshi et al. [9], we have not been able to replicate their results. They have similarly not been able to repeat the detection of SCARB2 on susceptible cells by flow cytometry (Satoshi Koike, personal communication, 2023); however, on mouse L929 cells transiently expressing human SCARB2, they did detect cell surface SCARB2 after biotinylation of the cell surface, pull down of biotinylated proteins and immunoblotting [22]. We saw no reduction in virus attachment when SCARB2 was knocked out with CRISPR/Cas9, a result consistent with a recent report by Guo et al. [47], who also found that EV-A71 is internalized in SCARB2-deficient cells. These results do not undercut a variety of strong evidence indicating that SCARB2 is important for EV-A71 infection [9,29], but they do indicate that SCARB2 functions primarily at a post-attachment step in infection, most likely uncoating in an acidic endosomal compartment [24].

## Materials and methods

### Cells

Jurkat cells (RBC0806) were obtained from the Riken Cell Bank. RD (CCL-136), HeLa (CRM-CLL-2), HEp-2 (CLL-23), 293T (CRL-3216), and Hep G2 (HB-8065) were purchased from the ATCC specifically for this study. RD-A cells, a derivative of RD cells with passage number 226, were provided by the Centers for Disease Control and Prevention, USA. These cell lines were authenticated with GenePrint 10 system (Promega) [57,58] by BEX Co., Ltd., Japan (S1 Data). Jurkat cells were cultured in Dulbecco’s modified Eagle’s medium (DMEM) without phenol red (Gibco, Cat# 21063–029) supplemented with ZellShield (Minerva Biolabs, Cat# 13–0050) and 10% fetal bovine serum (FBS) for EV-A71-EGFP infection or maintained in RPMI-1640 (Sigma Aldrich, Cat# R8758) supplemented with 10% FBS. RD, RD-A, and 293T cells were maintained in DMEM (Sigma Aldrich, Cat# D5796 or Fujifilm Wako, Cat# 040–30095) supplemented with 10% FBS (Sigma Aldrich, Cat# 172012, Lot# 15B377). HeLa, HEp-2, and Hep G2 cells were maintained in Eagle’s minimum essential medium (Fujifilm Wako, Cat# 051–07615) medium supplemented with 10% FBS. The human primary cells and the necessary culture reagents were purchased from ScienCell Research Laboratories and cultured according to the manufacturer’s instructions. Briefly, dermal fibroblasts-neonatal (Cat# 2310, Lot# 6445) and intestinal fibroblasts (Cat# 2920, Lot# 19238) were cultured in fibroblast medium (Cat# 2301) on poly-L-lysine (Cat# 0413) coated flasks, multi-well plates, or chambers. Tonsil endothelial cells (Cat# 2550, Lot# 18394) were cultured in endothelial cell medium (Cat# 1001) on bovine plasma fibronectin (Cat# 8248) coated flasks, multi-well plates, or chambers.

### Primary Abs

The mouse anti-CD162 (PSGL-1) mAb (clone KPL-1, Cat# 556052, Lot# 3225805) were purchased from BD Biosciences. The mouse IgG_1_ isotype control (clone MOPC-21, Cat# 400124, Lot# B136629) and IgG_2a_ (clone MOPC-173, Cat# 400202, Lot# B223740) were purchased from BioLegend. The goat anti-SCARB2 pAb (Cat# AF1966, Lot# KKY0115011 [Figs 5A, 5B and 8A], KKY0118021 [S1 and S7 Figs], KKY0118031 [Figs 8B, 9B, S2B and S6], KKY0121011 [Figs 2A, 3 and 9C]), normal goat IgG (Cat# AB-108-C, Lot# ES4119121, except for Fig 9C where Lot# ES4521041 was used), and the goat anti-human IgG Fc Ab (Cat# G-102-C, Lot# WBT1519101) were purchased from R&D Systems. The rabbit anti-SCARB2 mAb (clone 12H5L1, Cat# 702770, Lot# 2110715; clone 22H6L14, Cat# 703037, Lot# 2360697), rabbit IgG isotype control (Cat# 10500C, Lot# UA276761), the mouse anti-CD63 mAb (clone MEM-259, Cat# MA1-19281, Lot# WG3317432A), and the mouse anti-CD107a (LAMP-1) mAb (clone eBioH4A3, Cat# 14-1079-80, Lot# 2440973) were purchased from Invitrogen. The mouse anti-EEA1 mAb (clone 3C10, Cat# M176-3MS, Lot# 003) was purchased from MBL.

### Recombinant proteins and compound

Soluble recombinant forms of human SCARB2 fused to the Fc region of human IgG_1_ (LIMPII/SR-B2 Fc, Cat# 1966-LM, Lot# HPW0312071 [S2B Fig], Lot# HPW0915012 [Figs 2B, 5A, S1 and S3B]) and SCARB1-Fc (SR-BI Fc, Cat# 8114-SRB, Lot# DGZC0217031) were purchased from R&D Systems. SCARB1-Fc was used as a negative control Fc protein.

### Modification of plasmids

For directional cloning using a *Cpo*I site [59], we modified the expression plasmids as described below. The primers used for PCR are provided in S1 Table. The cDNA encoding blasticidin S deaminase was amplified from pEF6/V5-His A (Invitrogen) and cloned into the *Xba*I-*Not*I site of pEF1α-IRES (Clonetech) to produce pEF1α-IRES-bsd. The *Nhe*I-*Mlu*I fragment of pEF1α-IRES-bsd was replaced with the fragment containing a *Cpo*I site and EGFP gene amplified from pEGFP-N1 (Clonetech) by PCR to produce pEF1α-CpoI-EGFP-IRES-bsd. Then the *Nhe*I-*Not*I fragment of pEF1α-CpoI-EGFP-IRES-bsd was cloned into the *Spe*I-*Not*I site of pEF4/V5-His A (Clonetech) to produce pEF4-CpoI-EGFP-IRES-bsd. In the same way, we generated pEF4-CpoI-mCherry-IRES-bsd. On the other hand, the *Nhe*I-*Mlu*I fragment of pEF1α-IRES-bsd was replaced with the oligonucleotide containing a *Cpo*I site having the sequence 5′-gctagccggtccgaataatagtgaacgcgt-3′ (*Nhe*I-*Cpo*I-aa-Stop-Stop-Stop-*Mlu*I) to produce pEF1α-CpoI-IRES-bsd. Then the *Nhe*I-*Not*I fragment of pEF1α-CpoI-IRES-bsd was cloned into the *Spe*I-*NotI* site of pEF4/V5-His A (Clonetech) to produce pEF4-CpoI-IRES-bsd.

### Construction of expression plasmids

Human *SELPLG* cDNA encoding PSGL-1 was amplified by PCR from pEF-PSGL-1 plasmid [8]. The amplified product was digested with *Cpo*I and cloned into the *Cpo*I site of pEF4-CpoI-IRES-bsd to produce pEF4-PSGL-1-IRES-bsd. To prepare the control plasmid for transfecting Jurkat cells, the blasticidin S deaminase gene was amplified by PCR and cloned into the *Cpo*I site of pEF4-CpoI-IRES-bsd to produce pEF4-bsd-IRES-bsd. Human *SCARB2* cDNA was amplified by PCR from the cDNA of RD-A cells. The amplified product with a stop codon was digested with *Cpo*I and cloned into the *Cpo*I site of pEF4-CpoI-IRES-bsd to produce pEF4-SCARB2-IRES-bsd. The sequence of the cloned *SCARB2* open reading frame (ORF) was identical to that of *SCARB2* in GenBank (Accession No. NM_005506). The three mutations (N68Q, N325Q, and I476G) were introduced into *SCARB2* cDNA with PCR, and the mutated *SCARB2* cDNA was cloned into pEF4-CpoI-IRES-bsd to produce pEF4-SCARB2/QQG-IRES-bsd. The primers used for mutagenesis are provided in S2 Table. In the same way, human SCARB2 cDNA without a stop codon was amplified by PCR and cloned into pEF4-CpoI-EGFP-IRES-bsd and pEF4-CpoI-mCherry-IRES-bsd to produce pEF4-SCARB2-EGFP-IRES-bsd and pEF4-SCARB2-mCherry-IRES-bsd, respectively.

### Construction of the infectious cDNA clone of EV-A71

We used the EV-A71 strain SK-EV006 [39], which was used in the previous study [9], unless indicated. SK-EV006 is a PSGL-1-binding strain with glycine at amino acid position 145 of capsid protein VP1 [8,20]. Viral cDNA was generated as reported previously [20]. Full genomic cDNA fused to a T7 promotor sequence was amplified by PCR using the primers in S1 Table and cloned into the pBR322-derived plasmid, pBR322Y, as described previously [20]. Using the cloned SK-EV006 cDNA in pBR322Y as the template, the nucleotides different from those in the SK-EV006 plasmid sequence deposited by Yamayoshi et al. (GenBank accession No. AB469182) were fixed by site directed mutagenesis using PCR to replicate their experiments exactly [9]. The sequence in AB469182 had two mixed bases, y5250 and y5674. These two nucleotides were t5250 and t5674 in our plasmid. As these two nucleotides are in the genomic region encoding the non-structural proteins (3A and 3C, respectively), they would not be expected to affect the capsid-receptor interaction, and we left them unchanged. The resulting plasmid, pBREV71-SK-EV006 (sequence deposited as GenBank accession No. LC637980), is identical to the previously deposited sequence (GenBank accession No. AB469182) except for the two nucleotides at positions 5250 and 5674. We then inserted cDNA encoding EGFP, followed by the recognition sequence for viral protease 2A, just after the ATG codon of pBREV71-SK-EV006 to produce pBREV71-SK-EV006-EGFP, an infectious clone expressing EGFP, as described previously [9]. The viral cDNA sequence in pBREV71-SK-EV006-EGFP plasmid (GenBank accession No. LC637981) is identical to that in the EV71-GFP plasmid (GenBank accession No. AB469183) except for the two nucleotides at positions 5985 and 6409, which correspond to positions 5250 and 5674 in pBREV71-SK-EV006, respectively.

### Generation of viruses from the infectious viral cDNA clones

We generated infectious EV-A71-SK-EV006 virus from the pBREV71-SK-EV006 plasmid as described previously [20]. The four strains of EV-A71 (1095, 75-Yamagata, Nagoya, and 02363) were generated from pBREV71-1095-EG, pBREV71-75-Yamagata-EQ, pBREV71-Nagoya-EE, and pBREV71-02363-KE plasmids [20]. The viral RNA-transfected cells and supernatants were freeze-thawed three times at 24 h post-transfection. Before use in experiments, the recovered viruses were amplified in fresh RD-A cells. The EV-A71 expressing EGFP (EV-A71-EGFP) was generated from the pBREV71-SK-EV006-EGFP plasmid as described previously [21]. Cells were infected with EV-A71-EGFP in medium without phenol red (Gibco, Cat# 21063–029, 51200–038). The viral titers were determined by a microtitration assay using 96-well plates and RD-A cells. Briefly, 10 wells were used for each viral dilution. After adding RD-A cells, the plates were cultured for 7 days in a CO_2_ incubator. The viral titers were calculated by Spearman-Kärber method [60,61] and expressed as 50% cell culture infectious dose; CCID_50_. The viral RNA genome copies corresponding to 1 CCID_50_ were as follows: SK-EV006, 1.1 × 10^3^ copies; 1095, 1.5 × 10^3^ copies; 75-Yamagata, 3.1 × 10^2^ copies; Nagoya, 5.1 × 10^4^ copies; 02363, 1.3 × 10^3^ copies.

### Construction of CRISPR/Cas9 plasmids

To knock out the *SELPLG* and *SCARB2* genes, the CRISPR/Cas9 system was employed. pSpCas9(BB)-2A-Puro (PX459) V2.0 was a gift from Feng Zhang (Addgene plasmid # 62988; http://n2t.net/addgene:62988; RRID:Addgene_62988) [40]. First, the Cas9 gene in the plasmid was modified to increase fidelity [62]. Specifically, four mutations (N497A, R661A, Q695A, and Q926A) were introduced by site-directed mutagenesis to generate the PX459v2HF1 plasmid. The CRISPR/Cas9 oligonucleotides targeting the *SELPLG* and *SCARB2* genes were designed using CRISPRdirect [63]. The oligos cloned into PX459v2HF1 and plasmid names are provided in S3 Table.

### Establishment of Jurkat-PSGL-1-KO cells

Jurkat cells were transfected with PX459v2HF1-PSGL-1 by Amaxa cell line nucleofector kit V (Lonza). The cells cultured for a few weeks were stained with anti-PSGL-1 mAb followed by secondary Ab conjugated with Alexa Fluor 488. Then PSGL-1-negative cells were sorted by JSAN cell sorter (Bay bioscience) and cloned. The *SELPLG* genome editing was confirmed by PCR cloning and sequencing. Jurkat-PSGL-1-KO clone No. 1 harbored an insertion of “ggcc” between nucleotide position (np)133 and np134 (the number indicates the np of ORF) in one allele and a deletion of np133 to np145 (gaatatgagtacc) in another allele. Clone No. 2 harbored an insertion of “gagaggg” between np132 and np133 in one allele and a replacement of np123 to np130 (acaggcca) with a cytosine in another allele. For evaluation of the off-target effects of CRISPR/Cas9, Jurkat-PSGL-1-KO clones were transfected with pEF4-bsd-IRES-bsd or pEF4-PSGL-1-IRES-bsd. Then stable transfectants were selected with 2 μg ml^-1^ blasticidin S HCl (Wako, Cat# 029–18701). After selection, the cells (1 × 10^5^ cells) were inoculated with EV-A71-EGFP at 10 CCID_50_ per cell (250 μl in an 8-well chamber slide; Nunc, Cat# 154534) and incubated at 37°C for 12 h. Then EGFP expression was observed under a regular fluorescence microscope BZ-9000 (Keyence).

### Establishment of Jurkat-SCARB2-KO cells

Jurkat cells were transfected with PX459v2HF1-SCARB2 as described above. The cells were cloned and Jurkat-SCARB2-KO clones were selected by western blotting using anti-SCARB2 pAb. The *SCARB2* genome editing was confirmed by PCR cloning and sequencing. Jurkat-SCARB2-KO clone No. 1 harbored a replacement of np21 to np31 (cacggcgggga) with “gggc” in one allele and a deletion of np22 to np32 (acggcggggac) in another allele. Clone No. 2 harbored an insertion of a guanidine between np22 and np23 in one allele and a replacement of np21 and np22 (ca) with “agggtgtc” in another allele. For evaluation of the off-target effects of CRISPR/Cas9, Jurkat-SCARB2-KO clones were transfected with pEF4-mCherry-IRES-bsd or pEF4-SCARB2-mCherry-IRES-bsd. Then stable transfectants were selected with 2 μg ml^-1^ blasticidin S HCl. After selection, the cells (1 × 10^5^ cells) were inoculated with EV-A71-EGFP at 10 CCID_50_ per cell (250 μl in an 8-well chamber slide; Nunc, Cat# 154534) and incubated at 37°C for 12 h. Then expression of mCherry and EGFP was observed under a fluorescence microscope.

### Establishment of RD-SCARB2-KO cells

RD cells were transfected with PX459v2HF1-SCARB2 by Lipofectamine 3000 (Invitrogen). After 24 h transfection, medium was replaced with medium containing 5 μg ml^-1^ of puromycin to select transfected cells; the following day, this was replaced with normal medium. The cells were cloned and RD-SCARB2-KO clones were selected by western blotting using anti-SCARB2 pAb. The SCARB2 genome editing was confirmed by PCR cloning and sequencing. RD-SCARB2-KO clones No. 1 and No. 2 harbored a homozygous insertion of an alanine between np21 and np22. Clone No. 3 harbored the insertion of an alanine between np21 and np22 in one allele and a deletion of a cytosine at np23 in another allele. For evaluation of the off-target effects of CRISPR/Cas9, RD-SCARB2-KO clones were transfected with pEF4-EGFP-IRES-bsd or pEF4-SCARB2-EGFP-IRES-bsd. Then stable transfectants were selected with 10 μg ml^-1^ or 100 μg ml^-1^ blasticidin S HCl. After selection, the cells (1 × 10^5^ cells) were seeded in a well of an 8-well chamber slide (Matsunami, Cat# SCS-N08). Next day, EV-A71-SK-EV006 (2 × 10^6^ CCID_50_, MOI around 10 because cell number was almost doubled) was added to the well. The chamber slide was incubated with gentle agitation at room temperature (RT) for 30 min. Then the cells were cultured in a CO_2_ incubator at 37°C. The medium was replaced with 200 μl of fresh medium without phenol red 5 h after infection. The cells were cultured until 24 h post-infection. The development of cytopathic effects and EGFP expression were observed under a fluorescence microscope.

### Transfection of 293T cells

For flow cytometry and western blot, 293T cells were seeded at 6 × 10^5^ cells per 2.5 ml in a well of a 6-well plate 18 h before transfection and cultured in a CO_2_ incubator at 37°C. Four μg of plasmid was transfected according to the manufacturer’s instructions for Lipofectamine 2000 (ThermoFisher Scientific), except for using 10 μl of 2 mg ml^-1^ polyethyleneimine “MAX” (MW 40,000) (Polysciences) [64] instead of Lipofectamine 2000. The medium was replaced with 2.5 ml of fresh medium 4 h after transfection. The transfected cells were cultured until 24 h post-transfection and used for the experiments. For confocal microscopy, 293T cells were seeded at 3 × 10^4^ cells per 250 μl in a well of an 8-well cover glass chamber (Iwaki, Cat# 5232–008) 18 h before transfection. Zero point four μg of plasmid was transfected using 1 μl of 2 mg ml^-1^ polyethyleneimine as described above. The medium was replaced with 250 μl of fresh medium 4 h after transfection. The transfected cells were cultured until 24 h post-transfection and used for immune staining.

### Flow cytometry

Cells were detached with phosphate-buffered saline (PBS) (–) containing 5 mM EDTA (PBS-EDTA). The cells (2.5 × 10^5^ cells) were washed once with PBS-BSA (PBS (–) supplemented with 1% bovine serum albumin, 2 mM EDTA, and 0.1% NaN_3_) and incubated with an indicated Ab (0.25 μg per 25 μl) on ice for 10 min. After washing with PBS-BSA, the cells were stained with secondary Ab conjugated with Alexa Fluor 488 (50 μl of 1:500 dilution). The donkey anti-goat IgG (H+L) cross-adsorbed Ab conjugated with Alexa Fluor 488 (Invitrogen, Cat# A11055, Lot# 1942238) was used in Figs 2A, 5B, 9B and S6. The goat anti-rabbit IgG (H+L) superclonal Ab conjugated with Alexa Fluor 488 (Invitrogen, Cat# A27034, Lot# RD234895) was used in Figs 2A and 5B. The goat anti-mouse IgG (H+L) conjugated with Alexa Fluor 488 (Jackson Immuno Research, Cat# 115-545-062, Lot# 128726) was used in S3A and S4 Figs. Then the cells were incubated on ice for 10 min and washed twice with PBS-BSA. For SCARB2 detection, dead cells were stained with propidium iodide (Invitrogen, Cat# P3566; 2 μg ml^-1^ in PBS-BSA) at RT for 10 min. Then the propidium iodide-negative cells were gated to eliminate dead cells. Cells were analyzed by FACSCalibur and CellQuest Pro software (BD Biosciences).

### Western blot

Cells cultured in a well of a 6-well plate were washed twice with PBS (–), lysed with 200 μl of EzRIPA lysis buffer (Atto, Cat# WSE-742), and incubated on ice for 15 min. Then the cells were recovered in a 1.5-ml tube and centrifuged at 14,000 × g at 4°C for 5 min. The supernatant was recovered as the lysate. The protein concentration was measured by BCA protein assay kit (Takara, Cat# T9300A). The lysate in SDS-PAGE sample buffer was incubated at 95°C for 5 min. Ten μg of protein per lane was subjected to 10% or 12.5% SDS-PAGE. Then proteins were transferred onto Immobilon-FL membranes (Merck Millipore, Cat# IPFL20200). The membranes were blocked with 5% skim milk (Becton Dickinson, Cat# 232100) in PBS (–) with 0.05% Tween 20 (Sigma-Aldrich, Cat# P7949) and stained with Ab. The goat anti-SCARB2 pAb (R&D Systems, Cat# AF1966) was used at 0.1 μg ml^-1^ in Figs 5A and S1. The rabbit anti-SCARB2 mAb (clone 12H5L1) was used at a dilution of 1:1,000 in S3B Fig and 1:5,000 in Figs 2B, 5A and S1. The rabbit anti-SCARB2 mAb (clone 22H6L14) was used at a dilution of 1:5,000 in S1 Fig. The rabbit anti-goat IgG (H+L) superclonal Ab conjugated with HRP (Invitrogen, Cat# A27014, Lot# RK237620A, 1:10,000 dilution) was used in Fig 5A and S1. The goat anti-rabbit IgG (H+L) superclonal Ab conjugated with horseradish peroxidase (HRP) (Invitrogen, Cat# A27036, Lot# SE247011A, 1:200,000 dilution) was used in Figs 2B, 5A, S1 and S3B. In Fig 9A, the Lot# 2527613 was used at a 1:20,000 dilution. The goat anti-human IgG (Fcγ fragment specific) Ab conjugated with HRP (Cat# 109-035-098, Lot# 146365, 1:200,000 dilution) and the rabbit anti-goat IgG (Fcγ fragment specific) Ab conjugated with HRP (Cat# 305-035-046, Lot# 109300, 1:200,000 dilution) were purchased from Jackson Immuno Research and used in S2B Fig. Actin was detected by the rabbit anti-β-actin pAb conjugated with HRP (MBL, Cat# PM053-7, Lot# 006 [Fig 5A left], Lot # 009 [Figs 2B and S3B], 1:40,000 dilution) and the anti-actin pAb conjugated with HRP (Santa Cruz Biotechnology, Cat# sc-1615 HRP, Lot# L0205, 1:20,000 dilution, Fig 5A right). The immune complexes conjugated with HRP were visualized with SuperSignal West Femto Maximum Sensitivity Substrate (Thermo Scientific, Cat# 34095) or SuperSignal West Pico PLUS Chemiluminescent Substrate (Thermo Scientific, Cat# 34580) and were detected by using LAS-3000 image analyzer system (Fujifilm) or Amersham ImageQuant 800 (Cytiva). For stripping the immune complexes, the membrane was treated with WB stripping solution (Nacalai Tesque, Cat# 05364–55) for 5 min, washed, and blocked with 5% skim milk.

### Quantitative analysis of western blotting

The Fiji software [65] was used to quantify the intensity of signals in the western blot. The region of interested was selected using the rectangle tool, and then its mean gray value was measured. After subtracting the mean gray value of the background from the values of SCARB2 and actin, the adjusted mean gray value of SCARB2 was normalized by dividing it by that of actin. Finally, the relative amount of SCARB2 was calculated with that in RD cells being set as 1.

### EV-A71 infection assays of Jurkat cells

Cells (4 × 10^4^ cells) were inoculated with EV-A71-SK-EV006 at 1 CCID_50_ per cell on ice for 1 h, washed, and incubated in medium (200 μl in a 48-well plate) at 35°C. For Ab inhibition, the cells were pretreated with 10 μg ml^-1^ of anti-PSGL-1 mAb or 50 μg ml^-1^ of anti-SCARB2 pAb on ice for 1 h. Then EV-A71-SK-EV006 was added, incubated on ice for 1 h, washed, and maintained in medium without Ab. After 3 or 5 days, the infected cells and supernatants were freeze-thawed for three times and viral titers were determined by CCID_50_ titration by using RD-A cells. All infection assays were carried out in triplicate.

### EV-A71 infection assays of RD and RD-SCARB2-KO cells

Cells (1 × 10^5^ cells) were seeded in a well of 48-well plate. Next day, EV-A71-SK-EV006 (2 × 10^6^ CCID_50_, MOI around 10 because cell number was almost doubled) was added to the well. The plate was incubated with gentle agitation at 4°C for 30 min. Then the cells were washed three times and cultured in a CO_2_ incubator at 37°C. At the indicated time, the infected cells and supernatants were freeze-thawed for three times, and viral titers were determined by CCID_50_ titration by using RD-A cells. All infection assays were carried out in triplicate.

### EV-A71-EGFP infection assays

Jurkat cells (5 × 10^4^ cells) were inoculated with EV-A71-EGFP at 10 CCID_50_ per cell (200 μl in a 48-well plate), incubated at 37°C for 18 h, and fixed with 4% PFA phosphate buffer solution (Nacalai tesque, Cat# 09154–14). RD, HeLa, HEp-2, and 293T cells (2.5 × 10^4^ cells) were seeded in a well of 48-well plates. Hep G2 cells (1 × 10^6^ cells) were seeded in a well of 6-well plates. Next day, EV-A71-EGFP (5 × 10^5^ CCID_50_ for RD, HeLa, HEp-2, and 293T. 2 × 10^7^ CCID_50_ for Hep G2. MOI around 10 because cell number was almost doubled) was added. Then the plate was incubated in a CO_2_ incubator at 37°C for 18 h. The cells were then trypsinized, fixed with 4% PFA. The cells expressing EGFP were analyzed with FACSCalibur (BD Biosciences).

### EV-A71 detection by real-time RT-PCR

Viral RNA was detected by real-time RT-PCR as described previously by Johnsson et al. [66] with modifications. Five μl of viral RNA was assayed in a 20 μl reaction mixture using One step TB green PrimeScript plus RT-PCR kit (Takara, Cat# RR096A) with primers EnteroFw and EnteroRev [66] (final 1 μM each). The mixtures were subjected to real-time RT-PCR, consisting of 42°C for 5 min, 95°C for 10 s, followed by 40 cycles of 95°C for 3 s and 60°C for 30 s. The results were analyzed with 7500 Fast real-time PCR System, QuantStudio 3, or QuantStudio 5 (Applied Biosystems). In vitro transcribed RNA of EV-A71-SK-EV006 was used for quantification of copy number.

### Evaluation of inhibitory effect of anti-SCARB2 pAb on EV-A71 binding to SCARB2-Fc

The binding assay for EV-A71 and Fc proteins [8] was employed with minor modification. Briefly, 10 μl of Dynabeads protein A (Invitrogen, Cat# 10002D) and 0.2 μg of SCARB2-Fc were diluted in 100 μl of PBS (–) with 0.02% Tween 20 (PBS-T) and incubated at RT for 10 min. The beads were washed once with PBS-T and incubated with 5 μg of anti-SCARB2 pAb diluted in 100 μl of PBS-T at RT for 20 min. The beads were washed once. Then supernatant of infected RD-A cell culture (1 × 10^9^ copies of the EV-A71-SK-EV006 RNA genome) diluted in 100 μl of PBS-T was added and incubated on ice for 5 min. The beads were washed three times. A half of beads were suspended in 50 μl of distilled water with 10 μg ml^-1^ of yeast tRNA and incubated at 95°C for 5 min to release the virion RNA. Five μl of viral RNA was assayed by real-time RT-PCR in triplicate as described above. Another half of beads were suspended in 5 μl of SDS-PAGE sample buffer and heated at 95°C for 5 min. The supernatant was subjected to 10% SDS-PAGE and immunoblot to confirm anti-SCARB2 pAb binding to SCARB2-Fc. As a negative control for SCARB2-Fc, SCARB1-Fc was used. As SCARB2-Fc protein has human Fc region, goat anti-human IgG Fc Ab was also used as a negative control of inhibition.

### Evaluation of inhibitory effect of anti-SCARB2 pAb on EV-A71 binding to RD cells

Cells detached with PBS-EDTA (5 × 10^5^ cells) were treated with 50 μl of anti-SCARB2 pAb (50 μg ml^-1^) in PBS-BSA on ice for 30 min. Then the cells were pelleted by centrifugation and the supernatant was removed. The cells were suspended in 50 μl of PBS-BSA containing the supernatant of infected RD-A cell culture (4 × 10^8^ copies of the EV-A71-SK-EV006 RNA genome) and anti-SCARB2 pAb (50 μg ml^-1^) and incubated on ice for 30 min. Unbound virus was removed with three washes with PBS-BSA. Then viral RNA and cellular nucleic acids were extracted by High pure viral nucleic acid kit (Roche, Cat# 11858874001) according to the manufacturer’s instruction. At the final elution step, the nucleic acids were recovered in 80 μl of elution buffer. Then 5 μl of eluted RNA was assayed by real-time RT-PCR in a 20 μl reaction mixture in quintuplicate as described above. As an internal control for relative quantification (ΔΔCt), *ATP5F1* mRNA encoding ATP synthase peripheral stalk-membrane subunit b was detected by using human ATP5F1 primer set (Takara, Cat# HA035517).

### Evaluation of inhibitory effect of anti-SCARB2 pAb on EV-A71-EGFP infection

RD cells (2.5 × 10^3^ cells per 20 μl) were seeded in a well of CellCarrier-384 Ultra TC-treated plate (PerkinElmer, Cat# 6057302). Next day, the cells were pre-treated with goat anti-SCARB2 pAb or control goat Ab by adding 15 μl of diluted Ab (total volume: 35 μl) and incubating at 37°C for 30 min. Then EV-A71-EGFP (5 × 10^4^ CCID_50_, MOI around 10 because cell number was almost doubled) were added with the same final concentration of Ab in 15 μl of medium (total volume is 50 μl). Then the plate was incubated in a CO_2_ incubator at 37°C for 18 h. Finally, 50 μl of 10 μg ml^-1^ Hoechst 33342 (Invitrogen, Cat# H3570) were added and incubated at RT for 10 min. Fluorescence was detected and analyzed by Operetta CLS system with Harmony 4.9 software (PerkinElmer). All infection assays were carried out in quintuplicate and about 2 × 10^3^ cells per well were analyzed to calculate the percentage of EGFP-positive cells in cells stained with Hoechst 33342. DMEM without phenol red (Gibco, Cat# 21063–029) was used for these experiments. For human dermal fibroblasts (neonatal), intestinal fibroblasts, and tonsil endothelial cells, the cells (2.5 × 10^4^ cells) were seeded in a well of 48-well plates. Next day, the medium was removed and the cells were pre-treated with goat anti-SCARB2 pAb or control goat Ab by adding 150 μl of diluted Ab (50 μg ml^-1^) and incubating at 37°C for 30 min. Then EV-A71-EGFP (5 × 10^4^ CCID_50_, MOI around 10 because cell number was almost doubled) were added with 50 μg ml^-1^ of Ab in 50 μl of medium. Then the plate was incubated in a CO_2_ incubator at 37°C for 18 h. The cells were then trypsinized and fixed with 4% PFA. The cells expressing EGFP were analyzed with FACSCalibur (BD Biosciences).

### Evaluation of EV-A71 binding to RD and RD-SCARB2-KO cells

Cells detached with PBS-EDTA (5 × 10^5^ cells) were treated with 50 μl of PBS-BSA containing the supernatant of infected RD-A cell culture (4 × 10^8^ copies of the EV-A71-SK-EV006 RNA genome) and incubated on ice for 30 min. Unbound virus was removed with three washes with PBS-BSA. Then viral RNA and cellular nucleic acids were extracted and quantified by real-time RT-PCR as described above.

### Immunofluorescence microscopy for surface SCARB2

RD cells (3 × 10^4^ cells) were seeded in a well of an 8-well cover glass chamber (Iwaki, Cat# 5232–008) and cultured in a CO_2_ incubator at 37°C for two days. RD cells and transfected 293T cells were washed with ice-cold HBSS (Gibco, Cat# 14025–092) and then incubated with anti-SCARB2 mAb clone 22H6L14 (1 μg per 200 μl of HBSS) on ice for 30 min. Then the cells were washed and incubated with the donkey anti-rabbit IgG (H+L) highly cross-absorbed Ab conjugated with Alexa Fluor Plus 488 (Cat# A32790, Lot# UG288490, 0.2 μg per 200 μl of HBSS) with wheat germ agglutinin (WGA) conjugated with Alexa Fluor 633 (Invitrogen, Cat# W21404, Lot# 2160413, 1 μg per 200 μl) on ice for 30 min. The cells were washed on ice and fixed with 4% PFA at 37°C for 1.5 h. Finally, the cells were washed, treated with 10 μg ml^-1^ Hoechst 33342, and analyzed with a confocal microscope FV3000 (Olympus). The images were processed by Fiji to adjust the contrast/brightness according to the background fluorescence obtained by staining with negative control Ab and merge images [65].

### Immunofluorescence microscopy for intracellular SCARB2 and WGA

RD and RD-SCARB2-KO cells (3 × 10^4^ cells) were seeded and cultured as described above. The cells were washed with ice-cold HBSS and then incubated with WGA conjugated with Alexa Fluor 633 (1 μg per 200 μl of HBSS) on ice for 10 min. The cells were washed on ice and fixed with 4% PFA at 37°C for 20 min. Then the cells were washed twice and permeabilized by treatment with 0.4% Triton X-100 (Sigma-Aldrich, Cat# X100) in HBSS (HBSS-0.4T) at RT for 10 min. Then HBSS-0.4T was removed, and 10% normal donkey serum (Jackson Immuno Research, Cat# 017-000-121, Lot# 158760) diluted in 0.1% Triton X-100 in HBSS (HBSS-0.1T) was added for blocking. After 10 min incubation at RT, cells were washed once with HBSS-0.1T and incubated with anti-SCARB2 mAb clone 22H6L14 (1 μg per 200 μl of HBSS-0.1T) at RT for 1 h. Then the cells were washed twice with HBSS-0.1T and incubated with the donkey anti-rabbit IgG (H+L) highly cross-absorbed Ab conjugated with Alexa Fluor Plus 488 (Cat# A32790, Lot# UG288490, 0.2 μg per 200 μl of HBSS-0.1T) at RT for 30 min. Finally, the cells were washed three times with HBSS-0.1T, treated with 10 μg ml^-1^ Hoechst 33342, and analyzed with a confocal microscope as described above.

### Immunofluorescence microscopy for intracellular SCARB2 and organelle markers

RD cells (3 × 10^4^ cells) were seeded and cultured as described above. The cells were washed on ice and fixed with 4% PFA at 37°C for 20 min. Then the cells were washed twice and permeabilized by treatment with HBSS-0.4T at RT for 10 min. Then HBSS-0.4T was removed, and 10% normal donkey serum diluted in HBSS-0.1T was added for blocking. After 10 min incubation at RT, cells were washed once with HBSS-0.1T and incubated with anti-SCARB2 mAb clone 22H6L14 (1 μg per 200 μl of HBSS-0.1T) at RT for 1 h. Then the cells were washed twice with HBSS-0.1T and incubated with the donkey anti-rabbit IgG (H+L) highly cross-absorbed Ab conjugated with Alexa Fluor Plus 488 (Cat# A32790, Lot# UG288490) and the goat anti-mouse IgG (H+L) highly cross-absorbed Ab conjugated with Alexa Fluor Plus 647 (Cat# A32728TR, Lot# WE329964) (0.2 μg each per 200 μl of HBSS-0.1T) at RT for 30 min. Finally, the cells were washed three times with HBSS-0.1T, treated with 10 μg ml^-1^ Hoechst 33342, and analyzed with a confocal microscope as described above.

### Immunofluorescence microscopy by using goat anti-SCARB2 pAb

RD and RD-SCARB2-KO cells (3 × 10^4^ cells) were seeded in a well of an 8-well chamber (Matsunami, Cat# SCS-N08) and cultured in a CO_2_ incubator at 37°C for three days. For Ab staining prior to fixation, the cells were incubated with anti-SCARB2 pAb (1 μg per 200 μl of HBSS) on ice for 30 min. Then the cells were washed twice with ice-cold HBSS and incubated with the donkey anti-goat IgG (H+L) cross-absorbed Ab conjugated with Alexa Fluor 488 (Cat# A11055, Lot# 1942238, 0.2 μg per 200 μl of HBSS) on ice for 30 min. The cells were then washed three times with ice-cold HBSS, fixed with 4% PFA at 37°C for 30 min. Finally, the cells were washed three times with HBSS. For Ab staining after fixation but without permeabilization, the cells were washed three times with pre-warmed HBSS and fixed with 4% PFA at 37°C for 15 min. Then the cells were washed twice with HBSS and 10% normal donkey serum diluted in HBSS was added for blocking. After 10 min incubation at RT, cells were incubated with anti-SCARB2 pAb (1 μg per 200 μl of HBSS) on ice for 30 min. The cells were then washed twice with HBSS and incubated with the secondary Ab (0.2 μg per 200 μl of HBSS) on ice for 30 min. Finally, the cells were washed three times with HBSS. For Ab staining after fixation and permeabilization, the cells were washed three times with pre-warmed HBSS and fixed with 4% PFA at 37°C for 15 min. Then the cells were washed twice with HBSS and treated with HBSS-0.4T at RT for 10 min. After removing HBSS-0.4T, 10% normal donkey serum diluted in HBSS-0.1T was added for blocking. After 10 min incubation at RT, cells were incubated with anti-SCARB2 pAb (1 μg per 200 μl of HBSS-0.1T) on ice for 30 min. Then the cells were washed twice with HBSS-0.1T and incubated with the secondary Ab (0.2 μg per 200 μl of HBSS-0.1T) on ice for 30 min. Finally, the cells were washed three times with HBSS-0.1T. The cells were observed with a regular fluorescence microscope and analyzed as described above.

### Quantitative analysis of the confocal images

To calculate Pearson’s correlation coefficient, the BIOP JACoP plugin with the Moments threshold of the Fiji software [65] was used. Three independent experiments were conducted. In each experiment, five pairs of images were obtained and analyzed for each staining.

### Statistical analysis

We compared the viral titers, EGFP-positive percentages, percentages of colocalization in the confocal images, relative virus binding, relative amount of SCARB2 in the western blot, and RNA copies using Dunnett’s (Figs 3, 4A, 4B, 8C, 8D, 9A and S8), Tukey’s (Figs 7, 8A, 9C, S2B and S9), or Sidak’s (Fig 8B) multiple comparisons tests (two-sided) by using GraphPad Prism 8 software. *P* values of < 0.05 were considered statistically significant (Fig 8B and 8D).

## Supporting information

S1 Fig Anti-SCARB2 Abs used in this study recognize SCARB2-Fc but not SCARB1-Fc. To confirm the specificity of anti-SCARB2 Abs, recombinant SCARB2-Fc (50 ng) was detected by western blotting. As a negative control, recombinant SCARB1-Fc (50 ng) was loaded. After detection with anti-SCARB2 Ab, the membrane was stripped, blocked, and stained again with anti-Fc Ab as a loading control. The figure is representative of three independent experiments.
(TIF)

S2 Fig Anti-SCARB2 pAb inhibits EV-A71 binding to SCARB2-Fc. (A) SCARB2-Fc was incubated with Dynabeads protein A, and the complex was treated with (1) Control Ab, (2) Anti-Fc, or (3) Anti-SCARB2 pAb. Control Ab (goat IgG) and anti-Fc were used as negative control of Ab inhibition. Then EV-A71 was added, and beads were isolated with a magnet. After washing, EV-A71 bound to SCARB2-Fc was measured by real-time RT-PCR. (B) EV-A71 bound to SCARB2-Fc. Western blots show inhibitor pAb and Fc-fused protein precipitated with Dynabeads protein A. SCARB1-Fc was used as a negative control fusion protein (Control Fc). The figure is representative of three independent experiments. Results are indicated as the mean and s.e. for three independent experiments. The log_10_-transformed values were statistically analyzed by Tukey’s multiple comparisons test.
(TIF)

S3 Fig Establishment of Jurkat-PSGL-1-KO and Jurkat-SCARB2-KO cells. PSGL-1 or SCARB2 were knocked out by CRISPR/Cas9 in Jurkat cells, and two each of clones were established. (A) Flow cytometric analysis by anti-PSGL-1 mAb. The solid line and the shaded area represent staining with anti-PSGL-1 mAb and control mouse IgG_1_, respectively, followed by Alexa Fluor 488-tagged secondary Ab. The figure is representative of three independent experiments. (B) Western blotting analysis by anti-SCARB2 mAb (clone 12H5L1). Recombinant SCARB2-Fc (1 ng) was loaded as a positive control. The figure is representative of three independent experiments.
(TIF)

S4 Fig PSGL-1 expression confers EV-A71 susceptibility on Jurkat-PSGL-1-KO cells. To eliminate the possibility of off-target effects of CRISPR/Cas9, PSGL-1 was stably re-expressed in Jurkat-PSGL-1-KO clones (No. 1 and No. 2). PSGL-1 expression was confirmed by a flow cytometry. The solid line and the shaded area represent staining with anti-PSGL-1 mAb and control mouse IgG_1_, respectively, followed by Alexa Fluor 488-tagged secondary Ab. The cells infected with EV-A71-EGFP for 12 h were observed under a fluorescence microscope for evaluation of the EGFP expression. The figure is representative of three independent experiments. Scale bars, 100 μm.
(TIF)

S5 Fig SCARB2 expression confers EV-A71 susceptibility on Jurkat-SCARB2-KO cells. To eliminate the possibility of off-target effects of CRISPR/Cas9, SCARB2-mCherry was stably re-expressed in Jurkat-SCARB2-KO clones (No. 1 and No. 2). As a negative control, mCherry was stably expressed in the cells. The cells infected with EV-A71-EGFP for 12 h were observed under a fluorescence microscope for evaluation of the mCherry and EGFP expression. The figure is representative of three independent experiments. Scale bars, 100 μm.
(TIF)

S6 Fig RD-A cells do not express SCARB2 on the cell surface. Flow cytometric analysis of RD-A cells used in [9]. RD-A cells was stained with anti-SCARB2 pAb, followed by Alexa Fluor 488-tagged secondary Ab. The solid line and the shaded area represent staining with anti-SCARB2 pAb and control Ab, respectively. Note that the solid line and the border of the shaded area are almost completely overlapped, indicating the absence of SCARB2 on the cell surface. As a positive control of SCARB2 staining, cells expressing surface SCARB2 were stained and analyzed in parallel whenever possible. The figure (RD-A cells at passage number 235) is representative of at least fifteen independent experiments.
(TIF)

S7 Fig Fixation prior to anti-SCARB2 pAb treatment causes non-specific intracellular staining in RD and RD-SCARB2-KO cells. RD and RD-SCARB2-KO (clone No. 3) cells were used. Staining with anti-SCARB2 pAb, fixation with 4% PFA, and permeabilization were performed in the combination and order as indicated above the top panels. Finally the cells were stained with Alexa Fluor-tagged secondary Ab and observed under a regular fluorescence microscope. The figure is representative of three independent experiments. Scale bars, 200 μm.
(TIF)

S8 Fig SCARB2 is not involved in EV-A71 binding to RD cells, irrespective of EV-A71’s PSGL-1 binding phenotype. EV-A71 with VP1-145G or VP1-145Q are the PSGL-1-binding (PB) phenotype. EV-A71 with VP1-145E is the PSGL-1-nonbinding (non-PB) phenotype. RD and RD-SCARB2-KO (clone No.3) cells were reacted with EV-A71 (4 × 10^8^ genome copies) on ice for 30 min. Then the cells were washed, and cellular and viral nucleotides were extracted. EV-A71 bound to the cell were analyzed by real-time RT-PCR by ΔΔCt method using *ATP5F1* mRNA as an endogenous control. As a technical control of detection of reduced copy number, quarter (1 × 10^8^ genome copies) and half (2 × 10^8^ genome copies) amount of EV-A71 was tested in parallel. The relative virus binding of RD cells reacted with 4 × 10^8^ genome copies of EV-A71 was expressed as 1. Results are indicated as the mean and s.e. for three independent experiments.
(TIF)

S9 Fig SCARB2 is necessary for viral replication, irrespective of EV-A71’s PSGL-1 binding phenotype. EV-A71 with VP1-145G or VP1-145Q are the PSGL-1-binding (PB) phenotype. EV-A71 with VP1-145E is the PSGL-1-nonbinding (non-PB) phenotype. RD and RD-SCARB2-KO (clone No.3) cells were infected with EV-A71 (MOI around 10) at 4°C for 30 min. Then the cells were washed three times. Viral titers were determined immediately after washing (0 h) and following two days of incubation (2 d). Results are indicated as the mean and s.e. for triplicate samples.
(TIF)

S10 Fig SCARB2 expression confers EV-A71 susceptibility on RD-SCARB2-KO cells. To eliminate the possibility of off-target effects of CRISPR/Cas9, SCARB2-EGFP was stably re-expressed in RD-SCARB2-KO clones (No. 1, No. 2, and No. 3). As a negative control, EGFP was stably expressed in the cells. The cells infected with EV-A71-SK-EV006 for 24 h were observed under a florescence microscope for evaluation of the EGFP expression and the appearance of cytopathic effects (Phase). The figure is representative of three independent experiments. Scale bars, 100 μm.
(TIF)

S1 Table Primers for PCR amplification. (PDF)

S2 Table Primers for substitution. (PDF)

S3 Table Plasmids and oligos for genome editing by CRISPR/Cas9. (PDF)

S1 Data Cell authentication. (PDF)
