# Supplementary figures and images for: Enterovirus A71 does not meet the uncoating receptor SCARB2 at the cell surface
Source: PLoS Pathog. 2024 Feb 15;20(2):e1012022. doi: 10.1371/journal.ppat.1012022 (PMC10901359; doi:10.1371/journal.ppat.1012022)

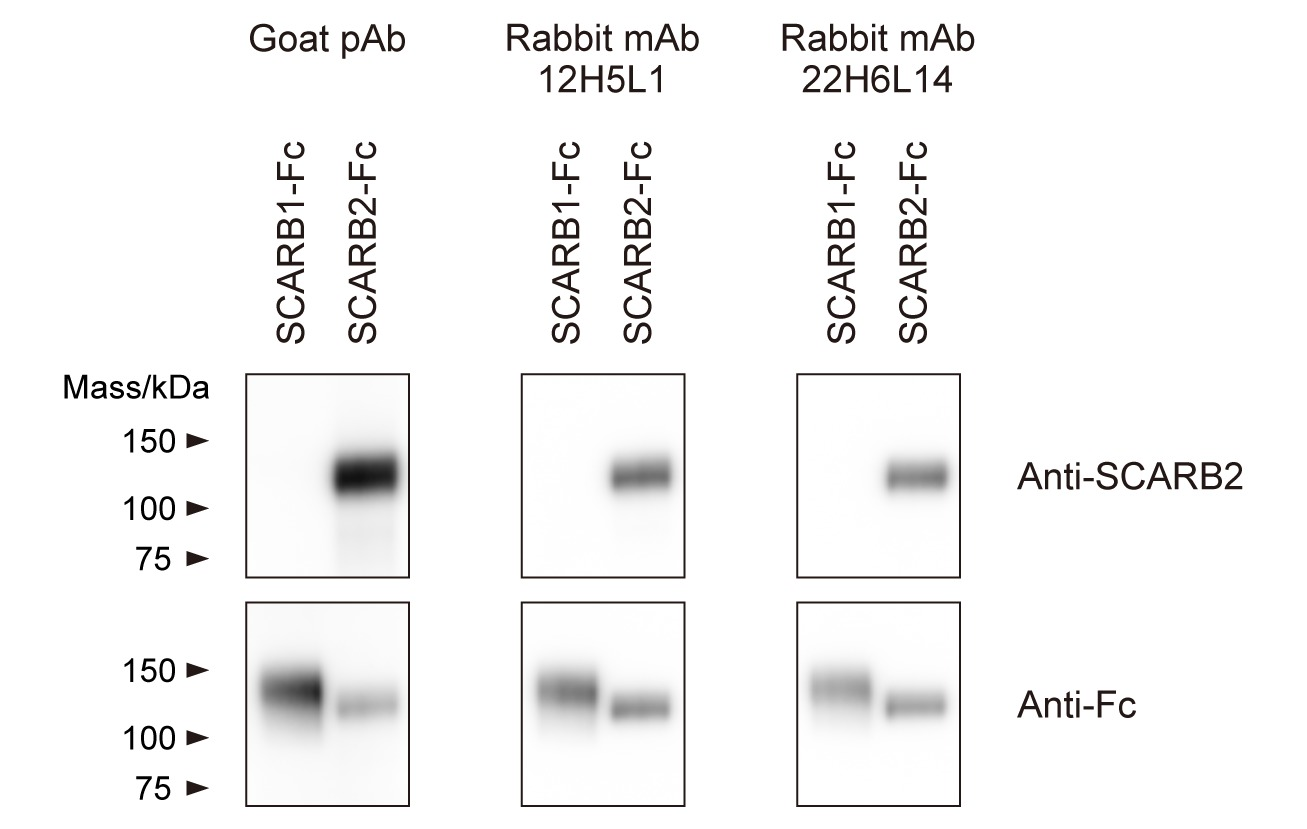

Supplement: S1 Fig — To confirm the specificity of anti-SCARB2 Abs, recombinant SCARB2-Fc (50 ng) was detected by western blotting. As a negative control, recombinant SCARB1-Fc (50 ng) was loaded. After detection with anti-SCARB2 Ab, the membrane was stripped, blocked, and stained again with anti-Fc Ab as a loading control. The figure is representative of three independent experiments. (TIF) [file ppat.1012022.s001.tif]

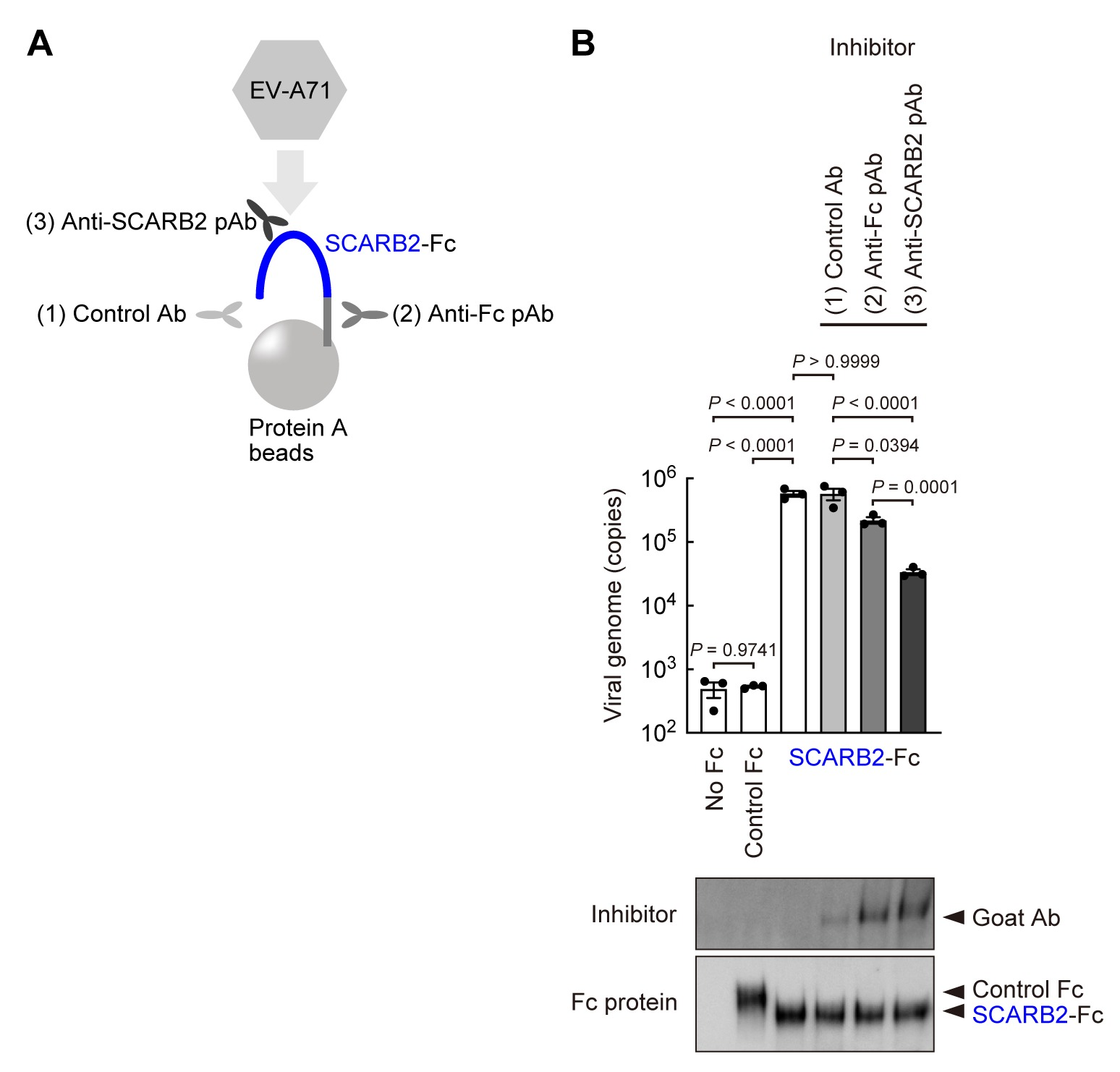

Supplement: S2 Fig — (A) SCARB2-Fc was incubated with Dynabeads protein A, and the complex was treated with (1) Control Ab, (2) Anti-Fc, or (3) Anti-SCARB2 pAb. Control Ab (goat IgG) and anti-Fc were used as negative control of Ab inhibition. Then EV-A71 was added, and beads were isolated with a magnet. After washing, EV-A71 bound to SCARB2-Fc was measured by real-time RT-PCR. (B) EV-A71 bound to SCARB2-Fc. Western blots show inhibitor pAb and Fc-fused protein precipitated with Dynabeads protein A. SCARB1-Fc was used as a negative control fusion protein (Control Fc). The figure is representative of three independent experiments. Results are indicated as the mean and s.e. for three independent experiments. The log10-transformed values were statistically analyzed by Tukey’s multiple comparisons test. (TIF) [file ppat.1012022.s002.tif]

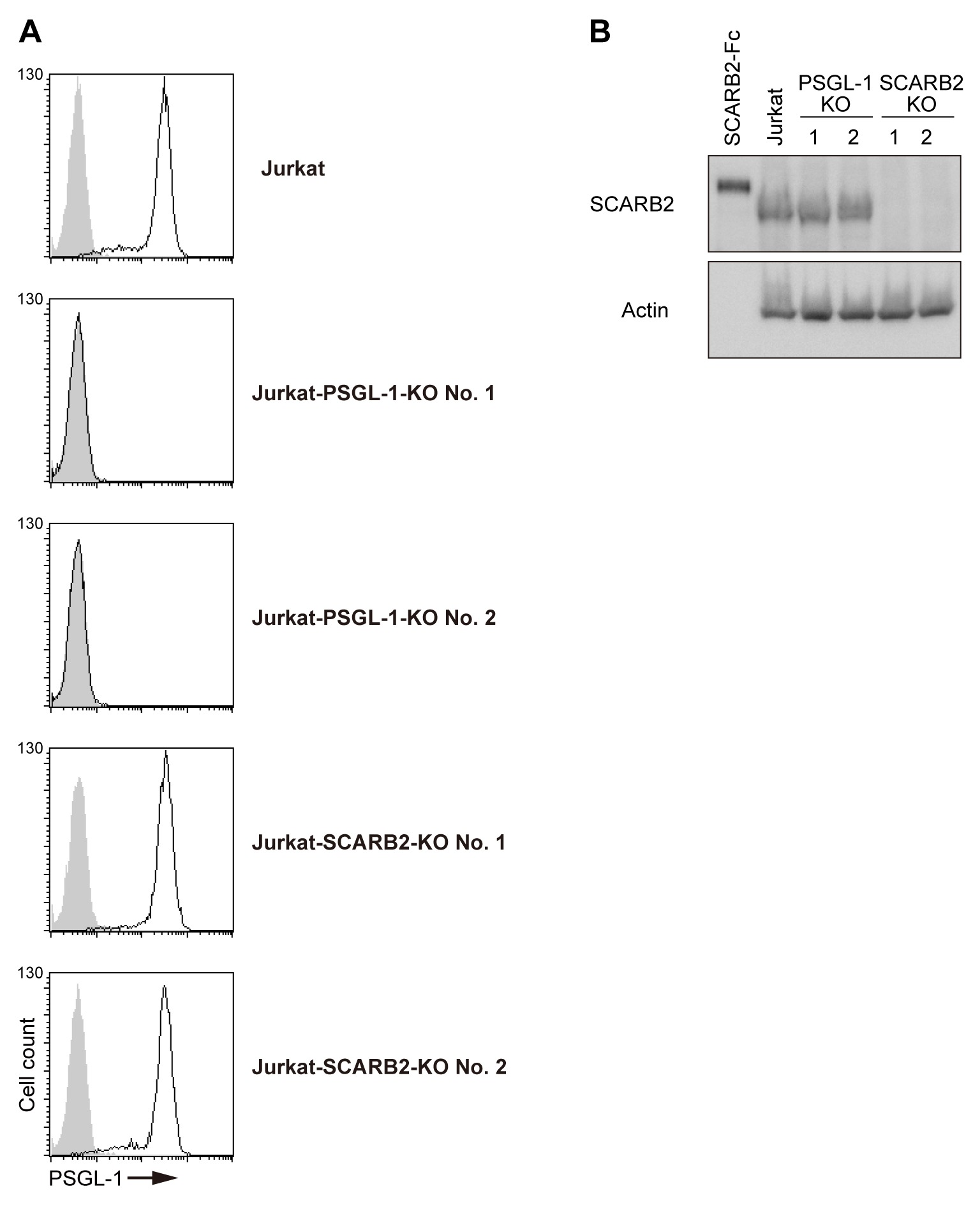

Supplement: S3 Fig — PSGL-1 or SCARB2 were knocked out by CRISPR/Cas9 in Jurkat cells, and two each of clones were established. (A) Flow cytometric analysis by anti-PSGL-1 mAb. The solid line and the shaded area represent staining with anti-PSGL-1 mAb and control mouse IgG1, respectively, followed by Alexa Fluor 488-tagged secondary Ab. The figure is representative of three independent experiments. (B) Western blotting analysis by anti-SCARB2 mAb (clone 12H5L1). Recombinant SCARB2-Fc (1 ng) was loaded as a positive control. The figure is representative of three independent experiments. (TIF) [file ppat.1012022.s003.tif]

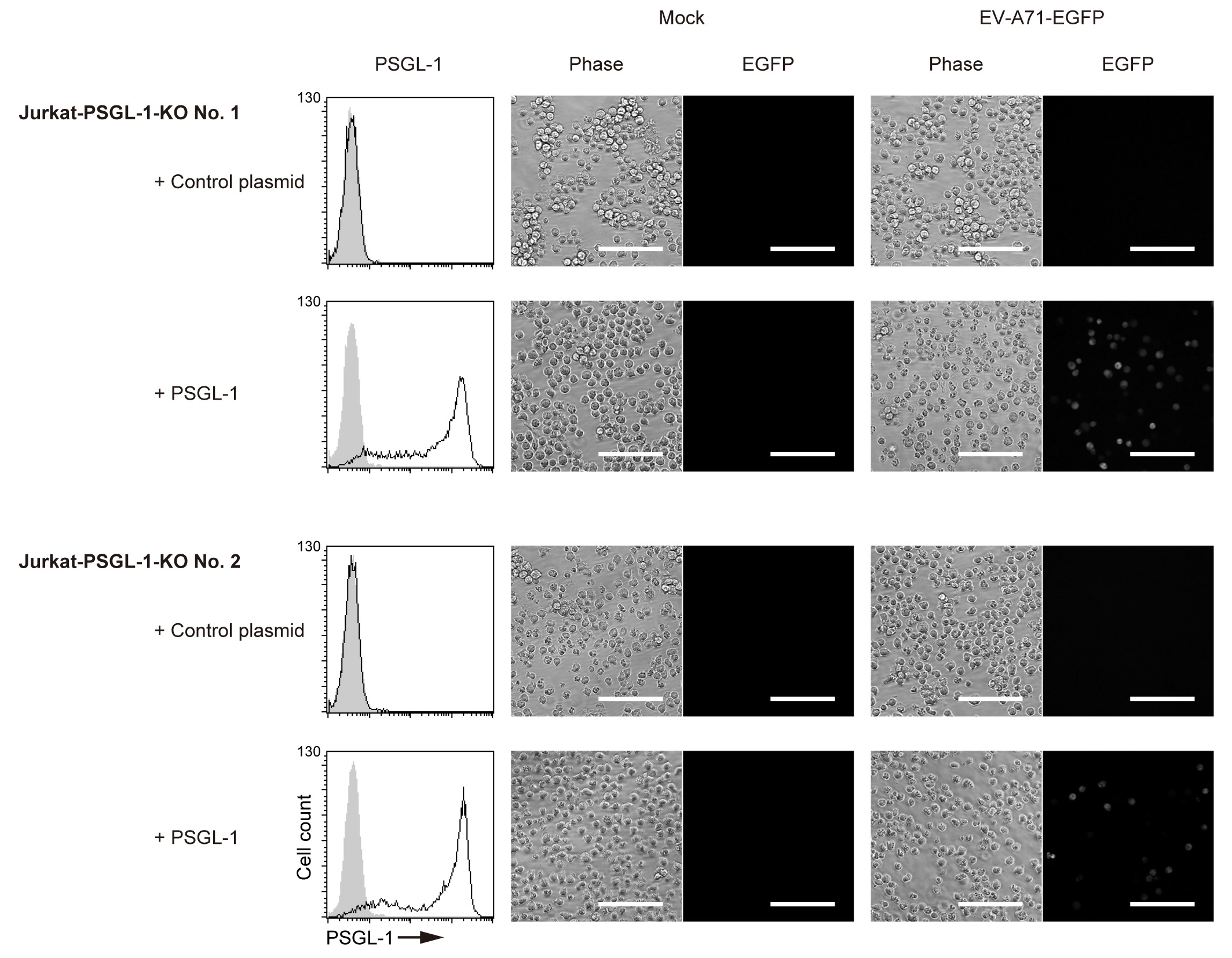

Supplement: S4 Fig — To eliminate the possibility of off-target effects of CRISPR/Cas9, PSGL-1 was stably re-expressed in Jurkat-PSGL-1-KO clones (No. 1 and No. 2). PSGL-1 expression was confirmed by a flow cytometry. The solid line and the shaded area represent staining with anti-PSGL-1 mAb and control mouse IgG1, respectively, followed by Alexa Fluor 488-tagged secondary Ab. The cells infected with EV-A71-EGFP for 12 h were observed under a fluorescence microscope for evaluation of the EGFP expression. The figure is representative of three independent experiments. Scale bars, 100 μm. (TIF) [file ppat.1012022.s004.tif]

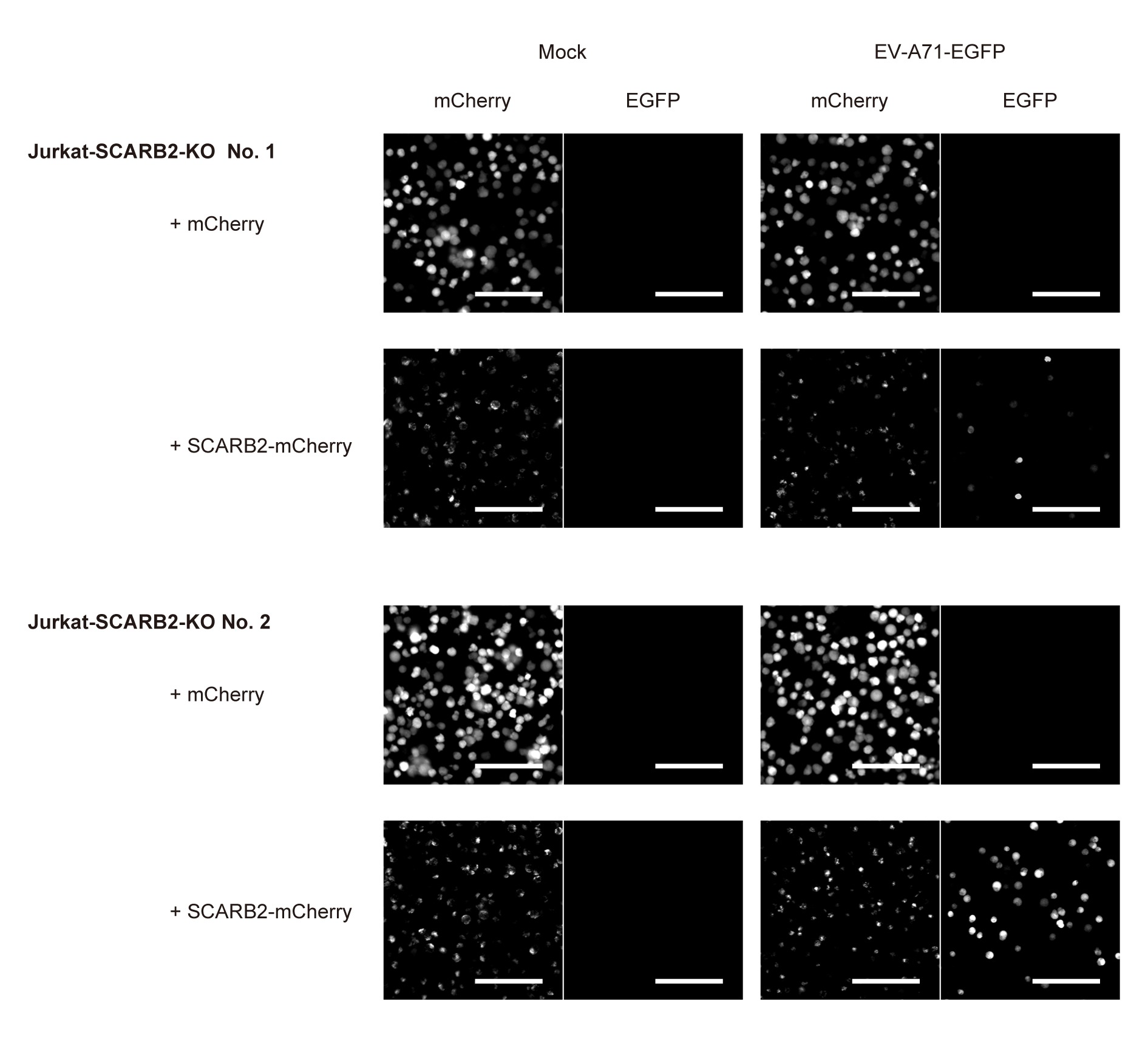

Supplement: S5 Fig — To eliminate the possibility of off-target effects of CRISPR/Cas9, SCARB2-mCherry was stably re-expressed in Jurkat-SCARB2-KO clones (No. 1 and No. 2). As a negative control, mCherry was stably expressed in the cells. The cells infected with EV-A71-EGFP for 12 h were observed under a fluorescence microscope for evaluation of the mCherry and EGFP expression. The figure is representative of three independent experiments. Scale bars, 100 μm. (TIF) [file ppat.1012022.s005.tif]

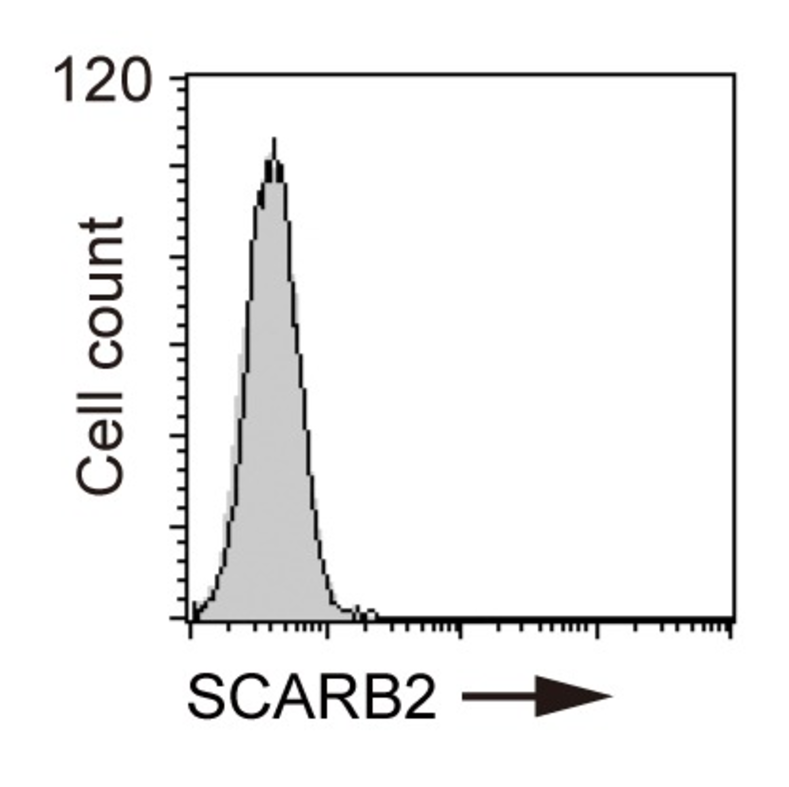

Supplement: S6 Fig — Flow cytometric analysis of RD-A cells used in [9]. RD-A cells was stained with anti-SCARB2 pAb, followed by Alexa Fluor 488-tagged secondary Ab. The solid line and the shaded area represent staining with anti-SCARB2 pAb and control Ab, respectively. Note that the solid line and the border of the shaded area are almost completely overlapped, indicating the absence of SCARB2 on the cell surface. As a positive control of SCARB2 staining, cells expressing surface SCARB2 were stained and analyzed in parallel whenever possible. The figure (RD-A cells at passage number 235) is representative of at least fifteen independent experiments. (TIF) [file ppat.1012022.s006.tif]

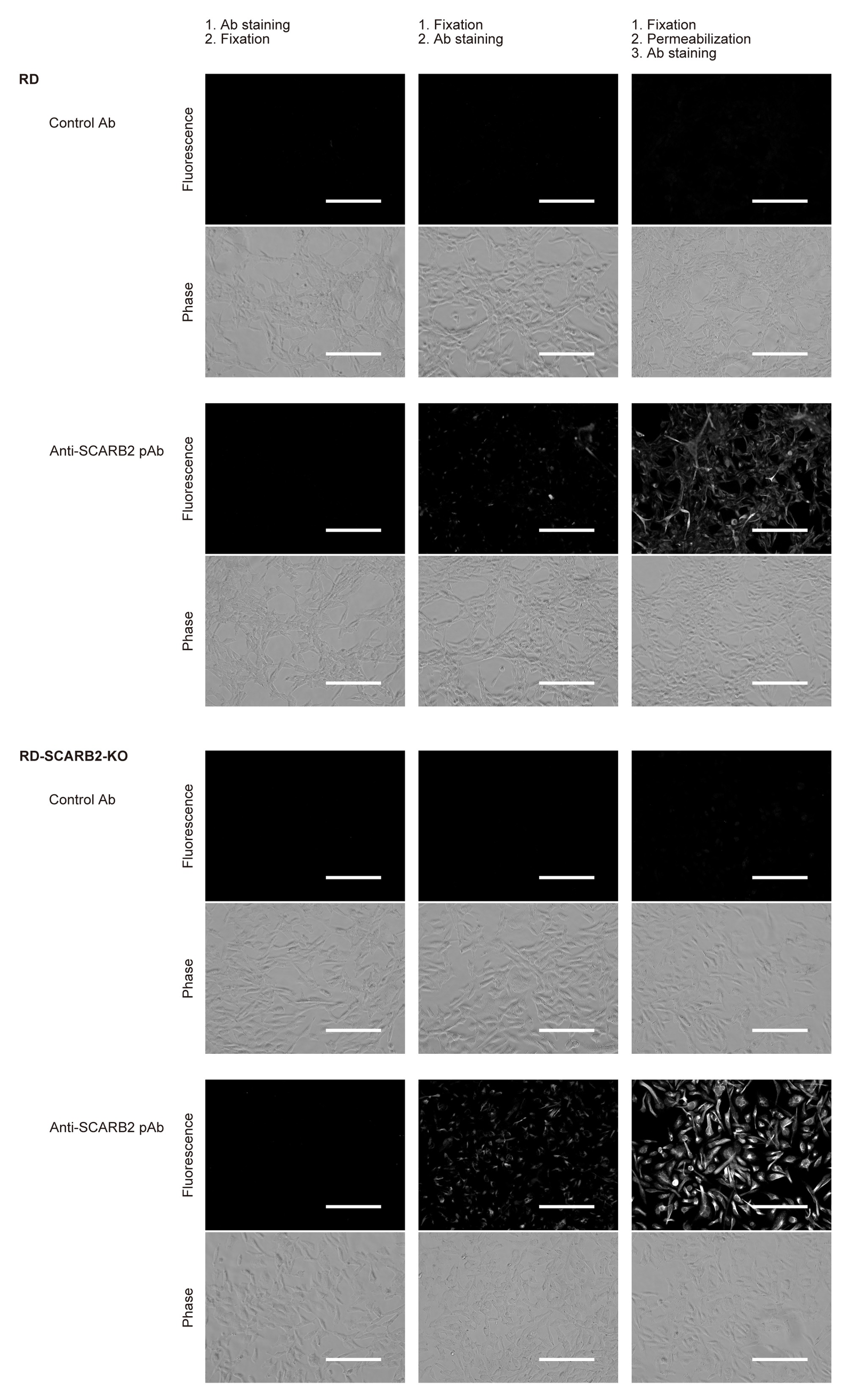

Supplement: S7 Fig — RD and RD-SCARB2-KO (clone No. 3) cells were used. Staining with anti-SCARB2 pAb, fixation with 4% PFA, and permeabilization were performed in the combination and order as indicated above the top panels. Finally the cells were stained with Alexa Fluor-tagged secondary Ab and observed under a regular fluorescence microscope. The figure is representative of three independent experiments. Scale bars, 200 μm. (TIF) [file ppat.1012022.s007.tif]

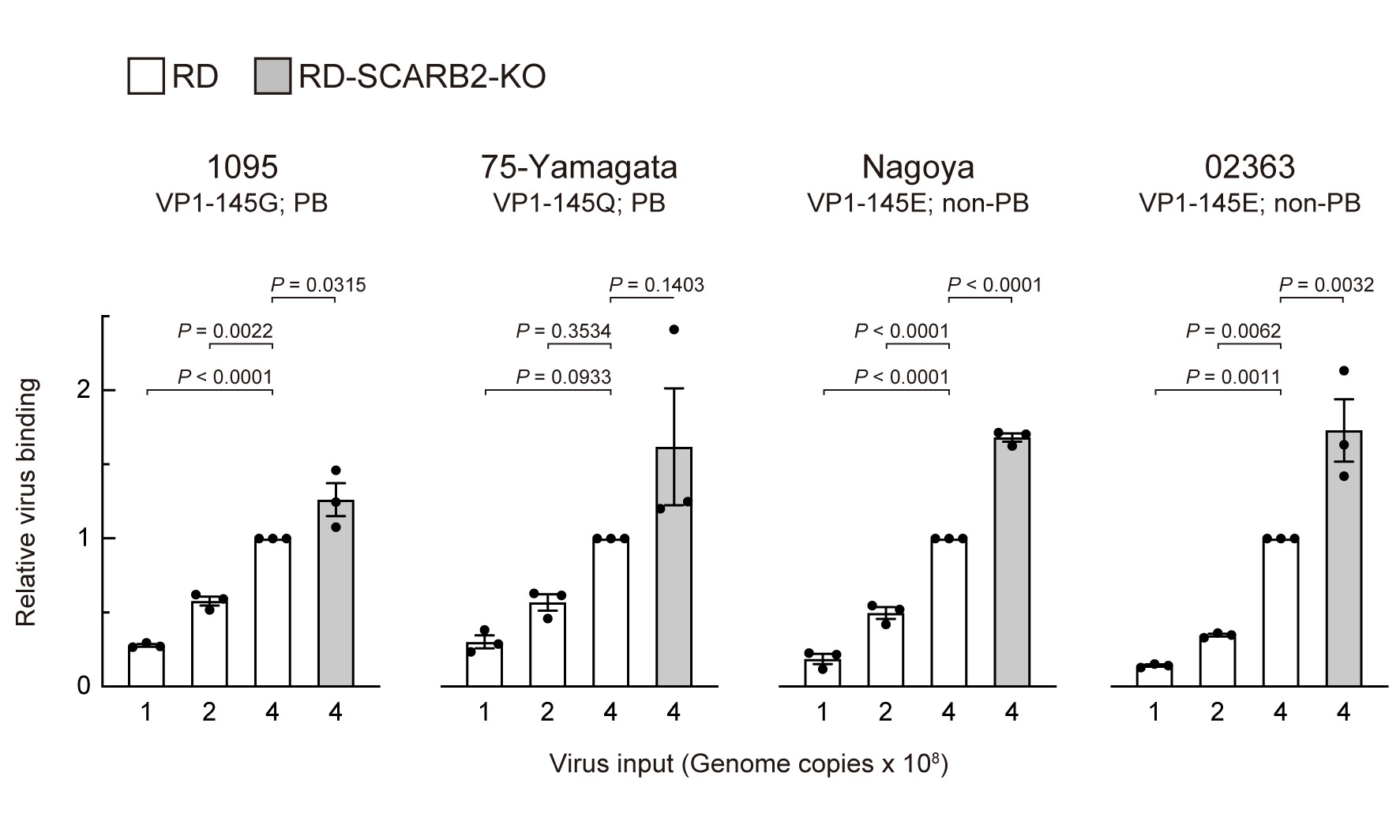

Supplement: S8 Fig — EV-A71 with VP1-145G or VP1-145Q are the PSGL-1-binding (PB) phenotype. EV-A71 with VP1-145E is the PSGL-1-nonbinding (non-PB) phenotype. RD and RD-SCARB2-KO (clone No.3) cells were reacted with EV-A71 (4 × 10^8^ genome copies) on ice for 30 min. Then the cells were washed, and cellular and viral nucleotides were extracted. EV-A71 bound to the cell were analyzed by real-time RT-PCR by ΔΔCt method using ATP5F1 mRNA as an endogenous control. As a technical control of detection of reduced copy number, quarter (1 × 10^8^ genome copies) and half (2 × 10^8^ genome copies) amount of EV-A71 was tested in parallel. The relative virus binding of RD cells reacted with 4 × 10^8^ genome copies of EV-A71 was expressed as 1. Results are indicated as the mean and s.e. for three independent experiments. (TIF) [file ppat.1012022.s008.tif]

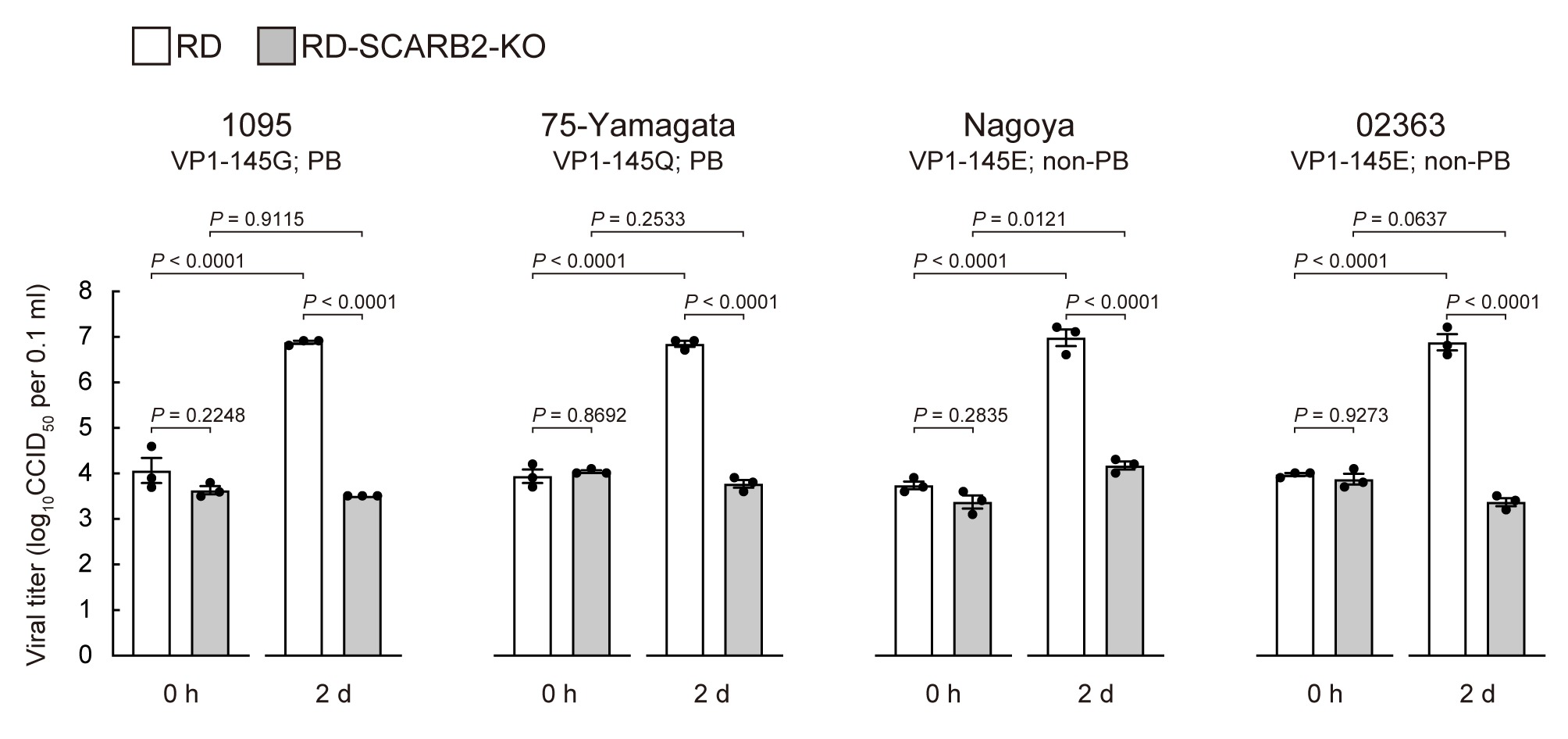

Supplement: S9 Fig — EV-A71 with VP1-145G or VP1-145Q are the PSGL-1-binding (PB) phenotype. EV-A71 with VP1-145E is the PSGL-1-nonbinding (non-PB) phenotype. RD and RD-SCARB2-KO (clone No.3) cells were infected with EV-A71 (MOI around 10) at 4°C for 30 min. Then the cells were washed three times. Viral titers were determined immediately after washing (0 h) and following two days of incubation (2 d). Results are indicated as the mean and s.e. for triplicate samples. (TIF) [file ppat.1012022.s009.tif]

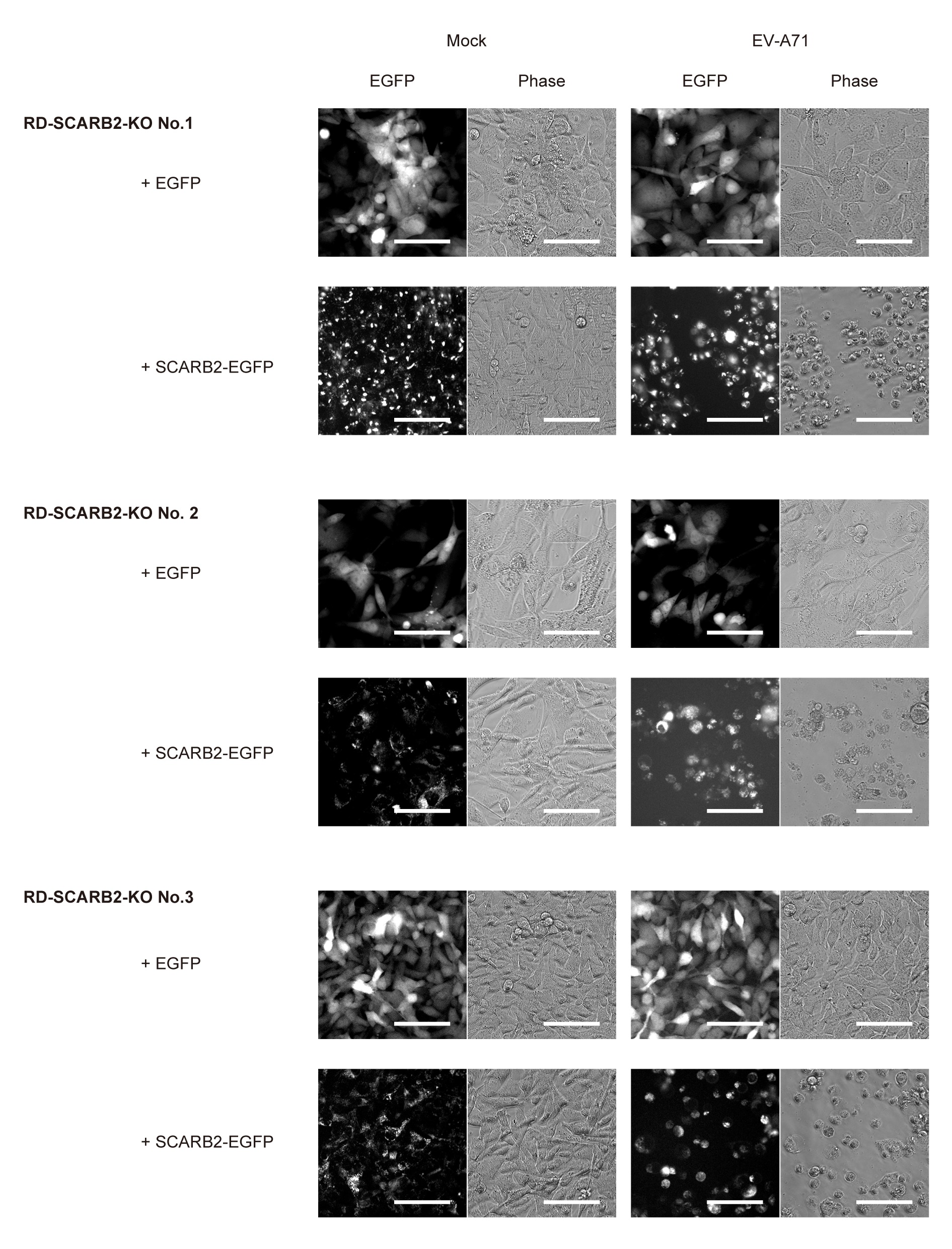

Supplement: S10 Fig — To eliminate the possibility of off-target effects of CRISPR/Cas9, SCARB2-EGFP was stably re-expressed in RD-SCARB2-KO clones (No. 1, No. 2, and No. 3). As a negative control, EGFP was stably expressed in the cells. The cells infected with EV-A71-SK-EV006 for 24 h were observed under a florescence microscope for evaluation of the EGFP expression and the appearance of cytopathic effects (Phase). The figure is representative of three independent experiments. Scale bars, 100 μm. (TIF) [file ppat.1012022.s010.tif]
