# Supplementary material for: Enterovirus A71 does not meet the uncoating receptor SCARB2 at the cell surface
Source: PLoS Pathog. 2024 Feb 15;20(2):e1012022. doi: 10.1371/journal.ppat.1012022 (PMC10901359; doi:10.1371/journal.ppat.1012022)
Supplement: S1 Table — (PDF) [file ppat.1012022.s011.pdf]

S1 Table. Primers for PCR amplification.

Construct	S/A[1)]	Site	Sequence (5′–3′)[2)]
EGFP, mCherry	S	*Nhe*I	tatagctagccggtccggaggtggaggtgccaccATGGTGAGCAAGGGCGAGGA
	A	*Mlu*I	tataacgcgtttaCTTGTACAGCTCGTCCA
Blasticidin S deaminase	S	*Xba*I	tctagaaccATGGCCAAGCCTTTGTC
	A	*Not*I	gcggccgcttaGCCCTCCCACACATAAC
	S	*Cpo*I	tatcggtccgccATGGCCAAGCCTTTGTCTCA
	A	*Cpo*I	tatcggaccgttaGCCCTCCCACACATAAC
PSGL-1	S	*Cpo*I	tatcggtccgataaatATGCCTCTGCAACTCCTCC
	A	*Cpo*I	tagcggaccgctaAGGGAGGAAGCTGTGCA
SCARB2	S	*Cpo*I	tatcggtccgataaatATGGGCCGATGCTGCTTCT
	A	*Cpo*I	taacggaccgttaGGTTCGAATGAGGGGTG (with a stop codon)
	A	*Cpo*I	acccggaccgGGTTCGAATGAGGGGTGCTC (without a stop codon)
EV-A71-SK-EV006	S	*Bam*HI	ttaggatcc*taatacgactcactata*ggttaaaacagcctgtgggttgcacccac
	A	*Stu*I	aaaaggccttttttttttttttttttttttttgctattctggttataacaaatttacccccac

[1)]S, sense; A, antisense

[2)]Restriction endonuclease recognition sites are underlined. Nucleotides corresponding to the open reading frame are indicated by uppercase letters. The T7 promoter sequence is indicated by italics.
