# Supplementary material for: Enterovirus A71 does not meet the uncoating receptor SCARB2 at the cell surface
Source: PLoS Pathog. 2024 Feb 15;20(2):e1012022. doi: 10.1371/journal.ppat.1012022 (PMC10901359; doi:10.1371/journal.ppat.1012022)
Supplement: S2 Table — (PDF) [file ppat.1012022.s012.pdf]

S2 Table. Primers for substitution.

Substitution	S/A[1)]	Sequence (5′–3′)[2)]
SCARB2 N68Q	S	ctatttcttcCaAgtcaccaatccagaggagat
	A	gattggtgacTtGgaagaaatagaactgagtat
SCARB2 N325Q	S	aggagttctgCaAgtcagcatctgcaagaatgg
	A	agatgctgacTtGcagaactcctgagcccaggc
SCARB2 I476G	A	taacggaccgttaggttcgaCCgaggggtgctctttcatccg (*Cpo*I)
Cas9 N497A	S	gatgaccGCcttcgataagaacctgcc
	A	atcgaagGCggtcatccgctcgatgaagc
Cas9 R661A	S	ctggggcGCCctgagccggaagctgat
	A	ggctcagGGCgccccagccggtgtatctcc
Cas9 Q695A	S	cttcatgGCCctgatccacgacgacagcct
	A	ggatcagGGCcatgaagtttctgttggcga
Cas9 Q926A	A	caggatctgtgccacgtgctttgtgatGGCccgggtttccaccagctgtc (*Pml*I)

[1)]S, sense; A, antisense.

[2)]Substituted nucleotides are indicated by uppercase letters. The restriction endonuclease recognition sites for cloning are underlined and indicated in parentheses
