# Supplementary material for: Enterovirus A71 does not meet the uncoating receptor SCARB2 at the cell surface
Source: PLoS Pathog. 2024 Feb 15;20(2):e1012022. doi: 10.1371/journal.ppat.1012022 (PMC10901359; doi:10.1371/journal.ppat.1012022)
Supplement: S3 Table — (PDF) [file ppat.1012022.s013.pdf]

S3 Table. Plasmids and oligos for genome editing by CRISPR/Cas9.

Plasmid	Target gene[1)]	S/A[2)]	Sequence (5′–3′)[3)]
PX459v2HF1-PSGL-1	*SELPLG* (131-150)	S	aaacCCGAATATGAGTACCTAGAT
		A	caccATCTAGGTACTCATATTCGG
PX459v2HF1-SCARB2	*SCARB2* (6-25)	S	caccCCGATGCTGCTTCTACACGG
		A	aaacCCGTGTAGAAGCAGCATCGG

[1)]The nucleotide positions corresponding to the open reading frame are indicated in parentheses.

[2)]S, sense; A, antisense.

[3)]Nucleotides used for cloning into the *Bbs*I site of PX459v2HF1 are indicated by lowercase letters. Nucleotides corresponding to the genome are indicated by uppercase letters.
