# Supplementary material for: Enterovirus A71 does not meet the uncoating receptor SCARB2 at the cell surface
Source: PLoS Pathog. 2024 Feb 15;20(2):e1012022. doi: 10.1371/journal.ppat.1012022 (PMC10901359; doi:10.1371/journal.ppat.1012022)
Supplement: S1 Data — (PDF) [file ppat.1012022.s014.pdf]

Sample : Jurkat (P3, 1/11/2022)

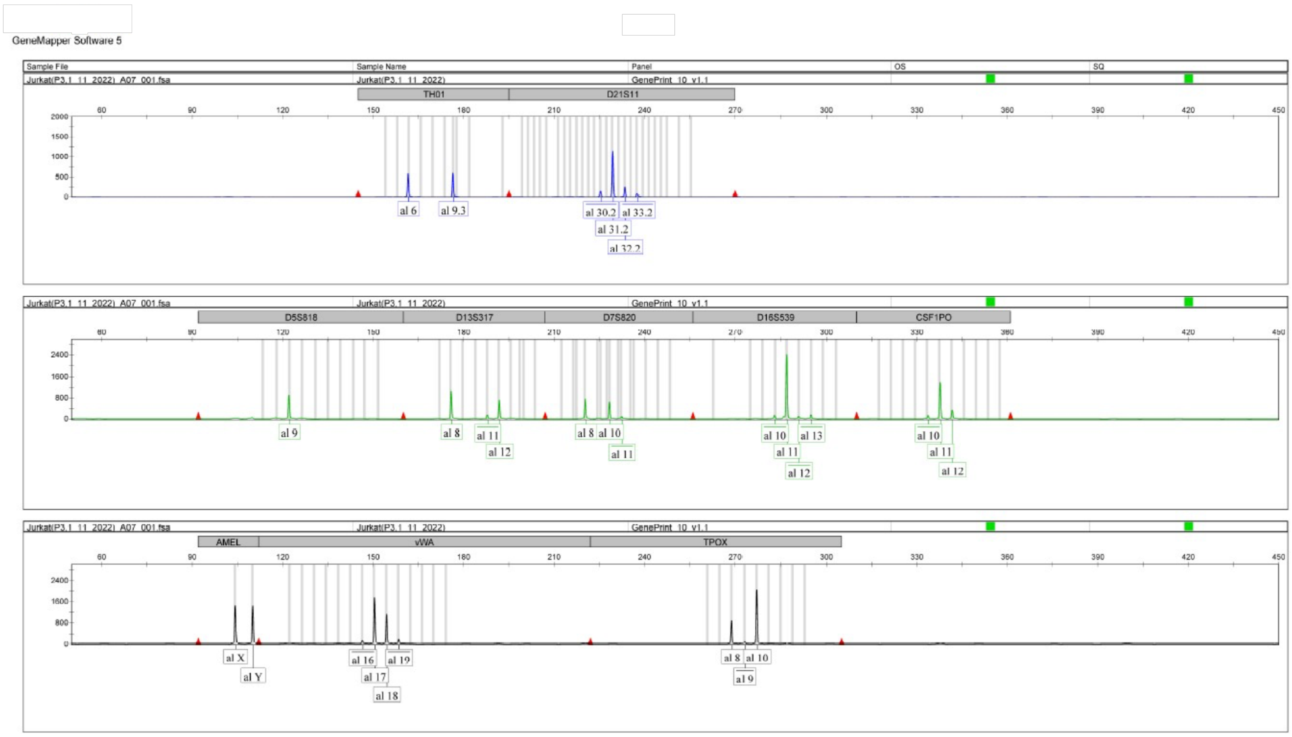

STR Profile

Locus	Jurkat (P3, 1/11/2022)			
TH01	6	9.3		
D21S11	30.2	31.2	32.2	33.2
D5S818	9			
D13S317	8	11	12	
D7S820	8	10	11	
D16S539	10	11	12	13
CSF1PO	10	11	12	
AMEL	X	Y		
vWA	16	17	18	19
TPOX	8	9	10	

Jurkat (CVCL_0065)		
6	9.3	
31.2	32.2	
9		
8	11	12
8	10	11
11		
11		
X	Y	
16	17	18
8	10	

STR profiles of Jurkat (P3, 1/11/2022) were not completely matched with Jurkat (CVCL_0065). But Evaluation value (EV) between Jurkat (P3, 1/11/2022) and Jurkat (CVCL_0065) was 0.82, which was high enough that STR profiles of Jurkat (P3, 1/11/2022) are the same as those of Jurkat (CVCL_0065).

Therefore two cell lines were considered to be the identical cell strain.

Sample：RD (P6, 9/4/2020)

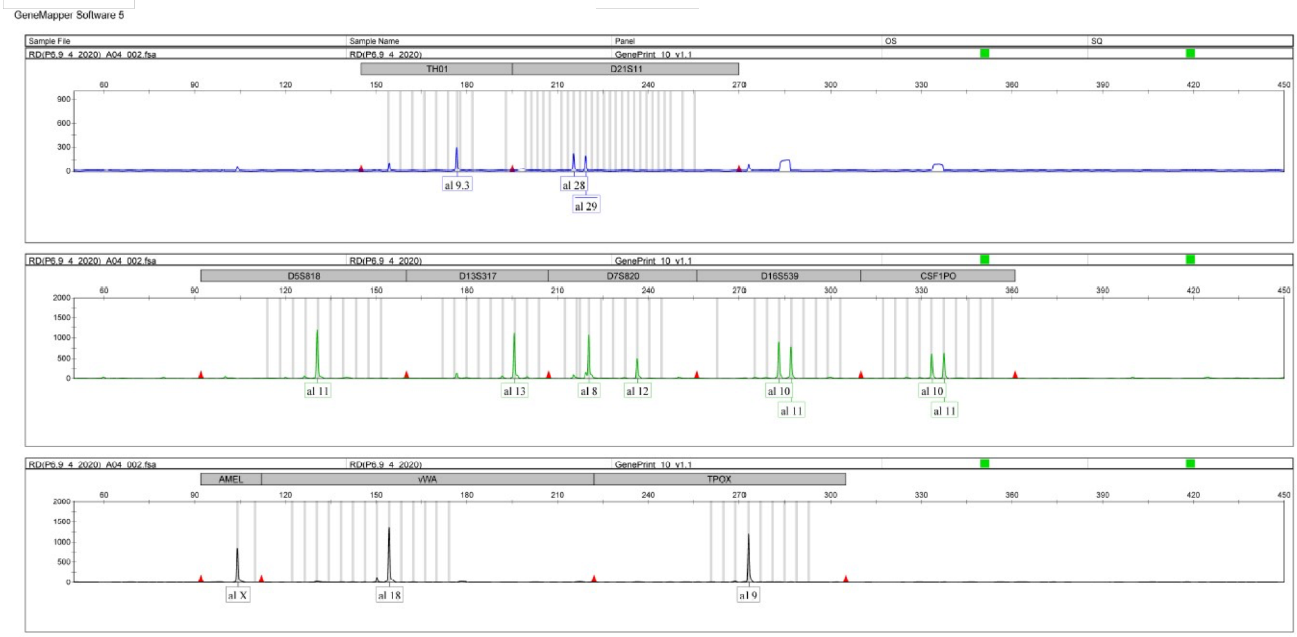

STR Profile

Locus	RD (P6, 9/4/2020)		RD (ATCC CCL-136)	
TH01	9.3		9.3	
D21S11	28	29	–	–
D5S818	11		11	
D13S317	13		13	
D7S820	8	12	8	12
D16S539	10	11	10	11
CSF1PO	10	11	10	11
AMEL	X		X	
vWA	18		18	
TPOX	9		9	

STR profiles of RD (P6, 9/4/2020) and RD (ATCC CCL-136) were completely matched. It was verified that the cells analyzed were considered to be the same as the cells registered in ATCC by comparison with the database of ATCC.

Sample：RD (P15, 10/9/2020)

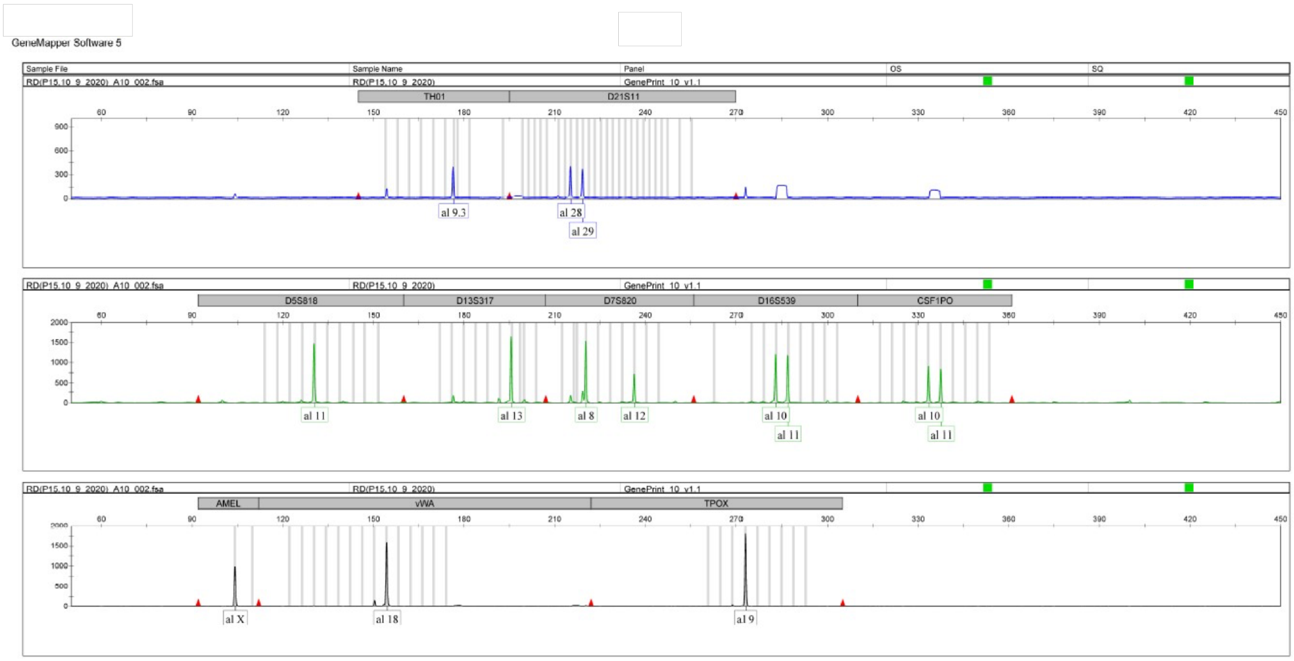

STR Profile

Locus	RD (P15, 10/9/2020)		RD (ATCC CCL-136)	
TH01	9.3		9.3	
D21S11	28	29	–	–
D5S818	11		11	
D13S317	13		13	
D7S820	8	12	8	12
D16S539	10	11	10	11
CSF1PO	10	11	10	11
AMEL	X		X	
vWA	18		18	
TPOX	9		9	

STR profiles of RD (P15, 10/9/2020) and RD (ATCC CCL-136) were completely matched. It was verified that the cells analyzed were considered to be the same as the cells registered in ATCC by comparison with the database of ATCC.

Sample：HeLa (P8, 9/16/2020)

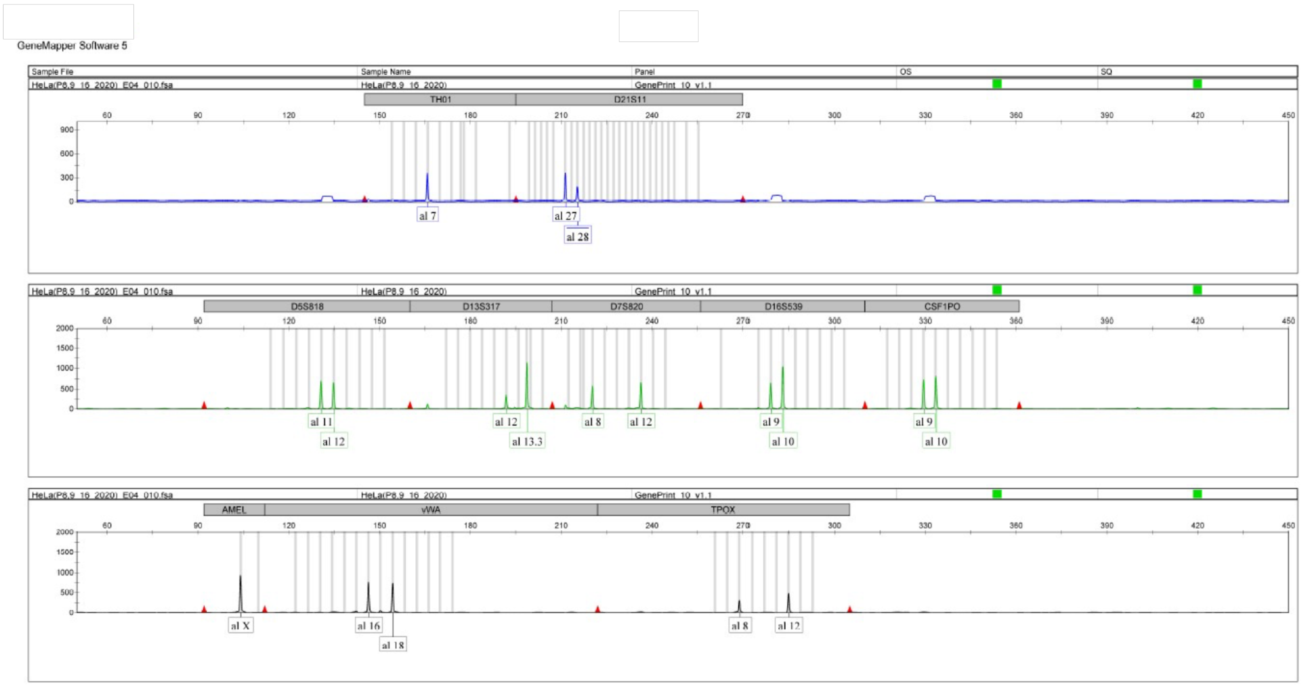

STR Profile

Locus	HeLa (P8, 9/16/2020)		HeLa (ATCC CRM-CCL-2)	
TH01	7		7	
D21S11	27	28	–	–
D5S818	11	12	11	12
D13S317	12	13.3	12	13.3
D7S820	8	12	8	12
D16S539	9	10	9	10
CSF1PO	9	10	9	10
AMEL	X		X	
vWA	16	18	16	18
TPOX	8	12	8	12

STR profiles of HeLa (P8, 9/16/2020) and HeLa (ATCC CRM-CCL-2) were completely matched. It was verified that the cells analyzed were considered to be the same as the cells registered in ATCC by comparison with the database of ATCC.

Sample：HEp-2 (P8, 9/16/2020)

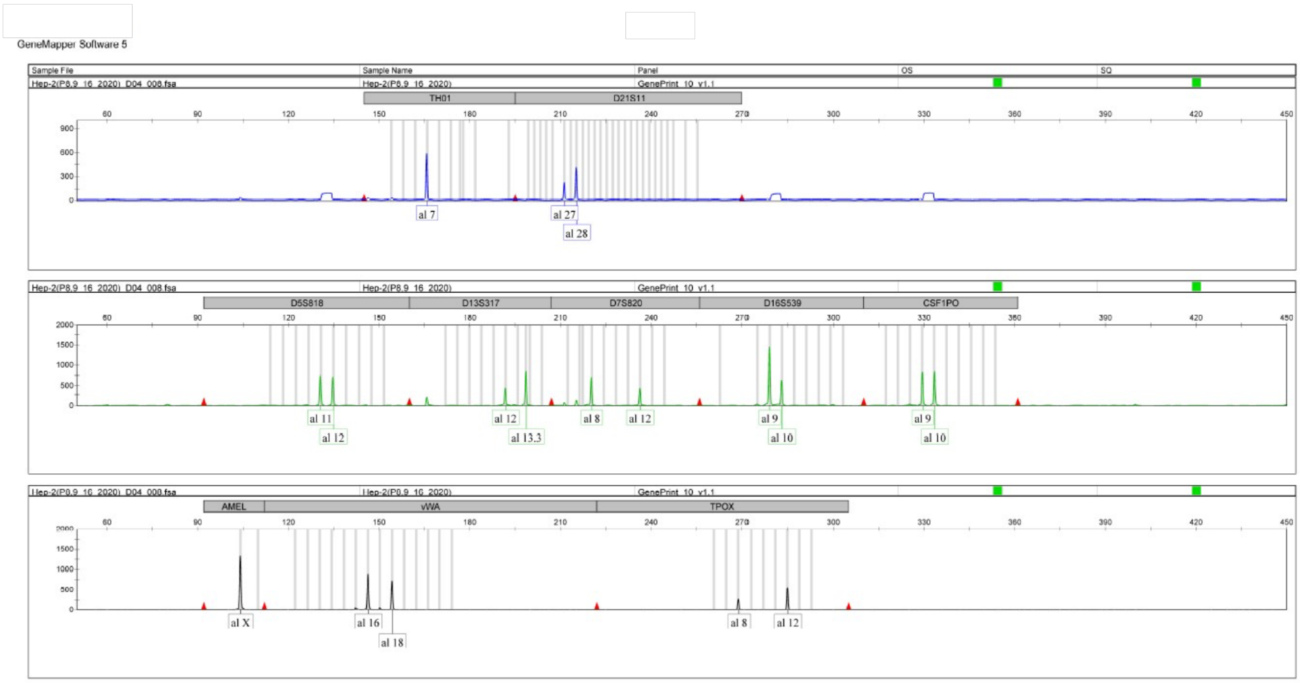

STR Profile

Locus	HEp-2 (P8, 9/16/2020)		HEp-2 (ATCC CCL-23)	
TH01	7		7	
D21S11	27	28	−	−
D5S818	11	12	11	12
D13S317	12	13.3	12	13.3
D7S820	8	12	8	12
D16S539	9	10	9	10
CSF1PO	9	10	9	10
AMEL	X		X	
vWA	16	18	16	18
TPOX	8	12	8	12

STR profiles of HEp-2 (P8, 9/16/2020) and HEp-2 (ATCC CCL-23) were completely matched. It was verified that the cells analyzed were considered to be the same as the cells registered in ATCC by comparison with the database of ATCC.

Sample：293T (P5, 9/16/2020)

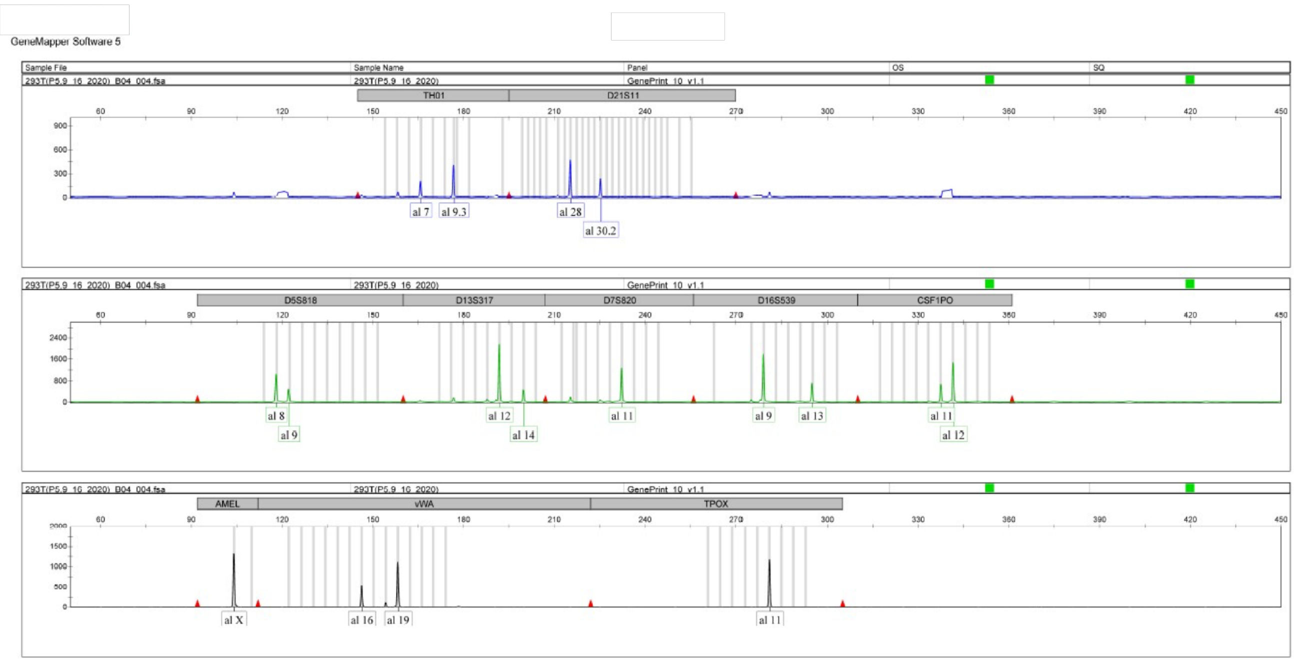

STR Profile

Locus	293T (P5, 9/16/2020)		293T (ATCC CRL-3216)	
TH01	7	9.3	7	9.3
D21S11	28	30.2	–	–
D5S818	8	9	8	9
D13S317	12	14	12	14
D7S820	11		11	
D16S539	9	13	9	13
CSF1PO	11	12	11	12
AMEL	X		X	
vWA	16	19	16	19
TPOX	11		11	

STR profiles of 293T (P5, 9/16/2020) and 293T (ATCC CRL-3216) were completely matched. It was verified that the cells analyzed were considered to be the same as the cells registered in ATCC by comparison with the database of ATCC.

Sample：Hep G2 (P8, 9/16/2020)

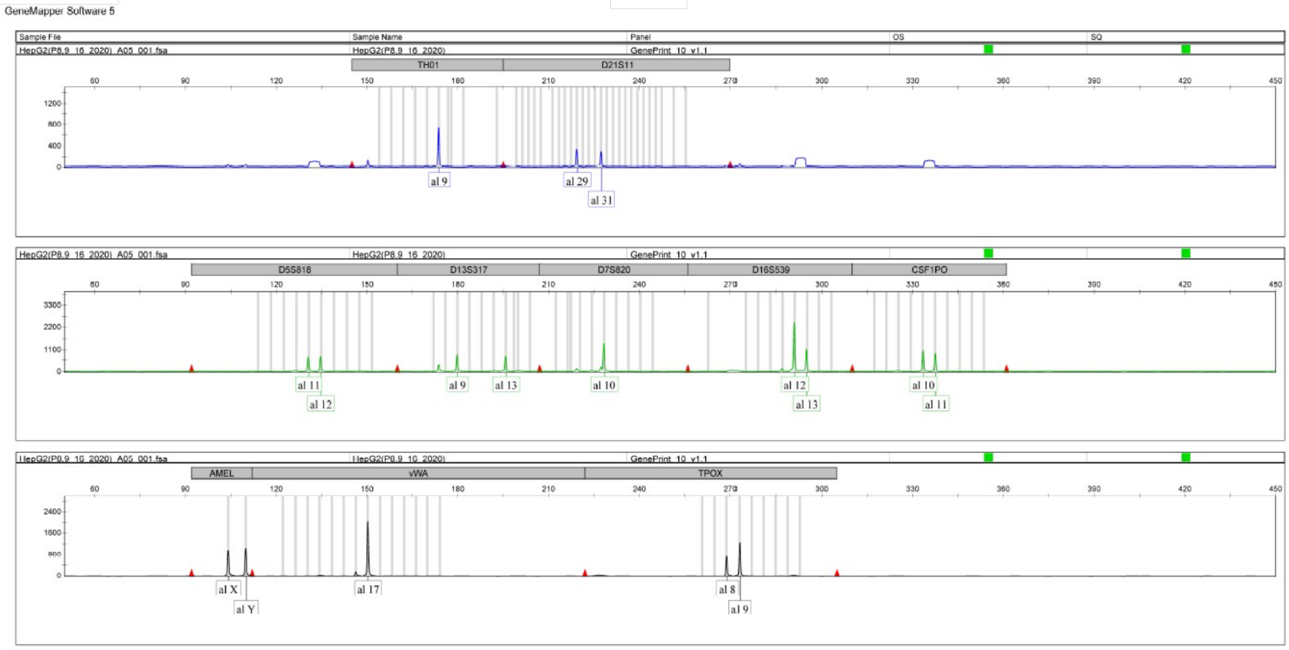

STR Profile

Locus	Hep G2 (P8, 9/16/2020)		Hep G2 [HEPG2] (ATCC HB-8065)	
TH01	9		9	
D21S11	29	31	–	–
D5S818	11	12	11	12
D13S317	9	13	9	13
D7S820	10		10	
D16S539	12	13	12	13
CSF1PO	10	11	10	11
AMEL	X	Y	X	Y
vWA	17		17	
TPOX	8	9	8	9

STR profiles of Hep G2 (P8, 9/16/2020) and Hep G2 [HEPG2] (ATCC HB-8065) were completely matched. It was verified that the cells analyzed were considered to be the same as the cells registered in ATCC by comparison with the database of ATCC.

Sample：RD-A (P226+2+4, 10/28/2020)

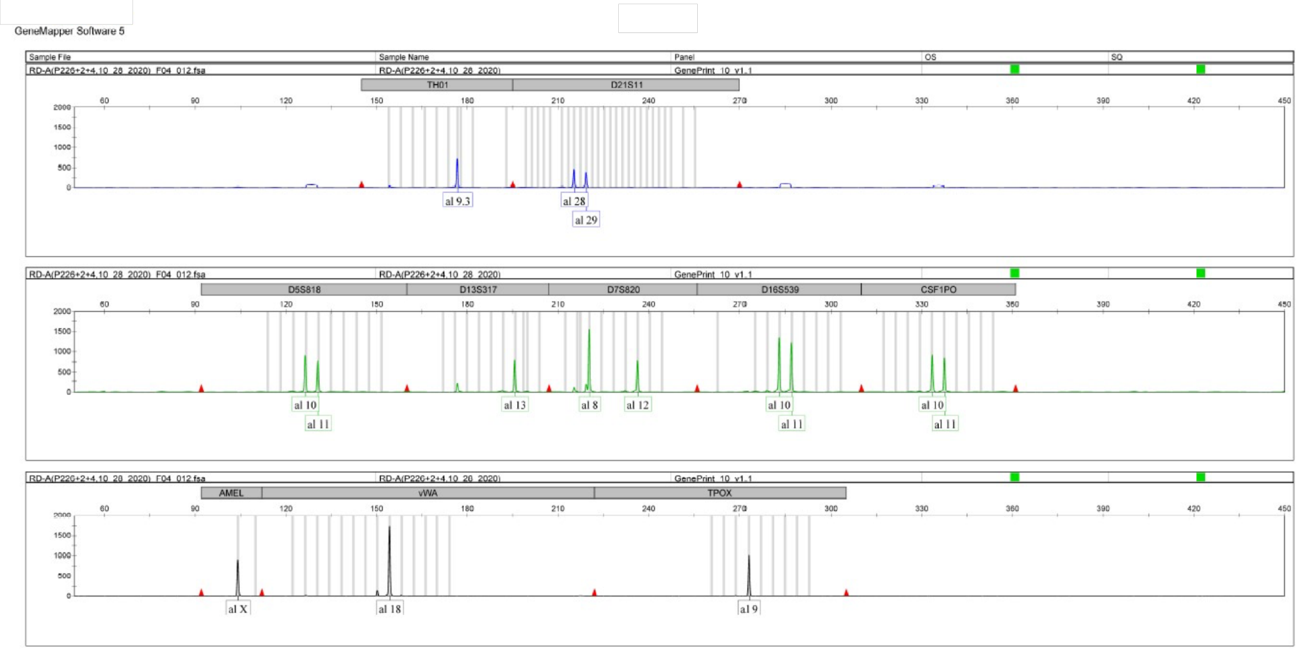

STR Profile

Locus	RD-A (P226+2+4, 10/28/2020)		RD (ATCC CCL-136)	
TH01	9.3		9.3	
D21S11	28	29	–	–
D5S818	10	11	11	
D13S317	13		13	
D7S820	8	12	8	12
D16S539	10	11	10	11
CSF1PO	10	11	10	11
AMEL	X		X	
vWA	18		18	
TPOX	9		9	

STR profiles of RD-A (P226+2+4, 10/28/2020) were not completely matched with RD (ATCC CCL-136). But Evaluation value (EV) between RD-A (P226+2+4, 10/28/2020) and RD (ATCC CCL-136) was 0.96, which was high enough that STR profiles of RD-A (P226+2+4, 10/28/2020) are the same as those of RD (ATCC CCL-136).

Therefore two cell lines were considered to be the identical cell strain.
